# Cigarette Smoke‐Induced Alveolar Macrophage Senescence via GEM/SIRT3‐Mediated Mitochondrial Dysfunction

**DOI:** 10.1002/advs.202522788

**Published:** 2026-06-22

**Authors:** Jin Wang, Meidan Wei, Xiangrong Song, Jiaxin Zhang, Lisha Zhang, Sixian Chen, Yaoyu Hu, Hui Gao, Jianxiang Li

**Affiliations:** ^1^ School of Public Health Suzhou Medical College of Soochow University Suzhou Jiangsu China

**Keywords:** alveolar macrophage senescence, GEM, N6‐methyladenosine, single‐cell transcriptomics, SIRT3

## Abstract

Smoking injury extends beyond the epithelium and endothelium; we show that alveolar macrophage senescence is a central driver. Using single‑cell RNA sequencing of human bronchoalveolar lavage fluid integrated with macrophage models exposed to cigarette smoke extract (CSE), we profiled senescence at the cell‑type level. The results of functional assays (mitochondrial reactive oxygen species (mitoROS), DNA damage, Senescence‐associated β‐galactosidase, p16/p21, apoptosis, phagocytosis, senescence‐associated secretory phenotype) confirmed the biology of these effects. Smokers’ macrophages were enriched for senescence and the SASP. In vitro, CSE increased mitoROS and DNA damage signaling, impaired phagocytosis, and induced apoptosis. Multiple cohort analyses revealed the GTP Binding Protein Overexpressed In Skeletal Muscle (GEM) as a causal driver: GEM was elevated in smokers and correlated with CDKN1A, and its perturbation altered the phenotype. GEM increased mitoROS, suppressed SIRT3/SOD2, lowered adenosine triphosphate (ATP), and amplified p16/p21 and SASP. Pharmacologic SIRT3 activation reversed these defects. In addition, the results from the mouse smoking model strongly support the role of the GEM/SIRT3 pathway in mediating the effects of cigarette smoke on macrophage senescence. Upstream, CSE induced ATF3 to transactivate GEM, while IGF2BP2 stabilized GEM mRNA via m^6^A. These data suggest that GEM–SIRT3 is an actionable biomarker and target for precision intervention in smoking‑related lung aging.

## Graphical abstract

Cigarette smoke accelerates the aging of immune cells in the lung. By combining human single‐cell sequencing, cell culture, and mouse models, the authors show that the protein GEM drives mitochondrial damage and senescence in alveolar macrophages by suppressing SIRT3. Reactivating SIRT3 reverses these defects, highlighting a druggable pathway in smoking‐related lung aging.

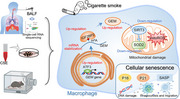

## 1 Introduction

Smoking is one of the most important and modifiable global risk factors for respiratory disease. It drives the onset and progression of chronic obstructive pulmonary disease (COPD), emphysema, and recurrent respiratory infections, leading to a substantial disease burden and premature mortality [1, 2, 3]. As sentinel cells at the airway‐alveolar interface, alveolar macrophages are directly exposed to complex chemical and particulate stressors in smoke. They clear particulates and pathogens and maintain immune homeostasis and tissue repair [4, 5]. Epidemiologic and experimental evidence has repeatedly demonstrated a causal link between smoking and lung injury. However, at the level of macrophages, the pathogenic nodes, druggable windows, and biological features that determine disease progression remain insufficiently defined [6, 7]. Smoking is closely associated with many aging‐related diseases, including various cancers, pulmonary fibrosis, and cardiovascular disease [3]. Senescent cells accumulate with age across tissues and drive age‐related pathology. They are therefore regarded as shared therapeutic targets for these conditions [8]. The concept of cellular senescence as an upstream driver of chronic inflammation and tissue remodeling has gained traction and offers a unifying framework for smoking‐related multiorgan injury [9, 10, 11].

Cellular senescence features persistent growth arrest, metabolic and chromatin reprogramming, and amplification of the senescence‐associated secretory phenotype (SASP), characterized by aberrant release of proinflammatory cytokines, chemokines, and proteases [11, 12]. The SASP engages in crosstalk with immune cells, disrupts the tissue microenvironment, and promotes fibrosis across multiple tissues, thereby driving disease initiation and progression [13]. Under smoke stress, diverse lung cell types—including epithelial, endothelial, and immune cells—exhibit senescence features [14, 15, 16]. In macrophages, the consequences are particularly consequential: impaired phagocytosis and pathogen clearance, a lowered inflammatory threshold with self‐sustaining activation, and increased tissue remodeling with damage to the elastic matrix [17, 18, 19]. These changes help explain smokers’ heightened susceptibility to infection and their increased risk of acute exacerbations. They may also drive irreversible structural damage to the lung parenchyma. However, how senescence signaling is initiated in macrophages—such as by smoke‐induced oxidative stress and DNA damage—and consolidated into a stable phenotype, whether a positive feedback loop forms with microenvironmental inflammation, and the extent to which these processes are reversible remain unclear.

Here, we employ a multilayer evidence chain spanning clinical samples and in vitro and in vivo models to elucidate the mechanisms of smoking‐induced macrophage senescence and to identify intervention strategies. Our goal is to inform precision interventions for smoking‐induced, aging‐related chronic diseases and to outline translational paths toward clinical application.

## 2 Methods

### 2.1 Participants and Grouping

We consecutively enrolled 20 adults (40–60 years) who were undergoing diagnostic bronchoscopy with bronchoalveolar lavage (BAL) for the evaluation of indeterminate pulmonary nodules at the Second Affiliated Hospital of Soochow University (Suzhou, Jiangsu Province, China). All participants were long‑term residents of the predominantly urban Suzhou metropolitan area. BAL samples were collected between July and August 2024. For the primary analyses, the subjects were grouped as smokers (n = 10) or nonsmokers (n = 10). To more precisely describe smoking exposure, participants were further categorized as current smokers, former smokers, or never smokers on the basis of standardized definitions (detailed in the Supporting Information). Basic demographic and clinical information (age, sex, indication for bronchoscopy, smoking history, sampling date, and BAL cell viability), as well as detailed inclusion and exclusion criteria, are provided in the Supporting Information. The study protocol was approved by the Institutional Review Board of the Second Affiliated Hospital of Soochow University (EC2024625), and written informed consent was obtained from all participants.

### 2.2 BAL Sample Collection and Initial Processing

Following standard bronchoscopy procedures, sterile normal saline (3 × 50 mL) was instilled slowly into the target segment (typically the right middle lobe or lingula). The fluid was immediately aspirated and pooled. Fifty milliliters of BAL fluid were used for this study. The samples were processed at 4 °C within 1–2 h. The cells were pelleted by centrifugation at 300 × g for 10 min at 4 °C, and the supernatant was discarded. Red blood cells were removed via red blood cell lysis buffer (BioLegend, #420301). After centrifugation, the pellet was resuspended in 1 mL of phosphate‐buffered saline (PBS). Cell counts and viability were assessed via trypan blue staining (Beyotime, # ST2780); samples with a viability ≥85% were subjected to downstream assays. The cells were aliquoted for flow cytometry or single‐cell RNA sequencing (scRNA‐seq) library preparation.

### 2.3 Flow Cytometry of BAL Cells

We used 1–2 × 10^6^ BAL fluid cells per assay. After Fc receptor blocking and exclusion of dead cells with a live/dead dye, the membranes were permeabilized via a fixation/permeabilization kit, followed by staining with anti‐human CD68 (BioLegend, # 333807) to identify the macrophages. The cells were incubated for 30 min at room temperature in the dark and washed with PBS. For live‐cell senescence‐associated β‐galactosidase (SA‐β‐gal) detection, we employed the C12FDG assay. Lysosomes were partially alkalinized with bafilomycin A1 (100 nm, 37°C, 1 h), followed by loading with C12FDG (APExBIO, # C8202) (33 µm, 37°C, 1 h). After washing with PBS, samples were acquired immediately. Data were collected on a BD FACSCanto II (Becton, Dickinson and Company, BD Biosciences), with at least 10 000 events per sample. Gating was performed in FlowJo v10 (BD Biosciences) as follows: FSC/SSC to exclude debris; FSC‐A/FSC‐H to select singlets; live cells; CD68+ cells; and C12FDG‐positive cells. The results are reported as the percentage of positive cells or mean fluorescence intensity (MFI).

### 2.4 Single‐Cell RNA Sequencing and Analysis

Library preparation and sequencing: Single‐cell libraries were prepared via the 10x Genomics Chromium Controller and single‐cell 3′ v3 or v3.1 chemistry according to the manufacturer's instructions. The target capture was 8000–10 000 viable cells per sample, with prelibrary viability ≥85%. Libraries passing Bioanalyzer quality control were sequenced on an Illumina NovaSeq 6000 (Agilent Technologies) via paired‐end reads (Read1 28 bp/Index 8 bp/Read2 91 bp), which targeted ≥50 000 reads per cell.

Primary processing and quality control: Cell Ranger (v8.0.1) was used to align reads to the human reference genome (GRCh38) and to generate count matrices. The matrices were imported into Seurat (v4) in R (v4.3). The cell‐level filters excluded cells with nFeature_RNA <200 or >6000 or a mitochondrial transcript proportion >15%. Cells with markedly elevated hemoglobin transcripts were flagged as potential erythrocyte contaminants and excluded from the sensitivity analyses. Doublets were identified and removed with DoubletFinder (v2.0.4). Ambient RNA contamination was corrected with SoupX (v1.6.2). The data were normalized with SCTransform (default parameters), the mitochondrial proportion and sequencing depth were regressed out, and the cell cycle scores were regressed when necessary to minimize their impact on clustering.

Integration, dimensionality reduction, and clustering: Donor‐level batch effects were corrected via Seurat integration anchors or Harmony. The top 30 principal components (PCs) were used for downstream analyses. Clustering employed the Louvain/Leiden algorithm (resolution 0.4–1.2, tuned for biological interpretability). UMAP was used for visualization.

### 2.5 Cell Lines and Culture

The human acute monocytic leukemia cell line THP‐1 (Procell, #CL‐0233) and the mouse alveolar macrophage line MH‐S (Procell, #CL‐0597) were maintained under standard conditions in a humidified incubator at 37°C with 5% CO_2_. The culture medium was RPMI‐1640 supplemented with 2 mm L‐glutamine and 10% fetal bovine serum (FBS).

Differentiation: THP‐1 cells were stimulated with phorbol 12‐myristate 13‐acetate (PMA, Solarbio, # P6741) at 100 ng/mL for 48 h. The PMA was then removed, and the cells were allowed to rest in fresh complete medium for 24 h. Successful differentiation was defined by an adherent phenotype, increased cell size, and upregulation of macrophage markers such as CD11b and CD68.

### 2.6 Preparation and Application of Cigarette Smoke Extract (CSE)

Mainstream smoke from commercially available cigarettes was bubbled at a constant flow, driven by a peristaltic pump, through two serial collection chambers. Each chamber contained 50 mL of serum‐free Dulbecco's Modified Eagle Medium (DMEM). One hundred percent CSE was generated by combusting five cigarettes at 5 min per cigarette. The extract was sterilized through a 0.22 µm filter, aliquoted, and stored at −80°C. The absorbance at 320 nm (A320) was used to monitor batch consistency. The working concentrations were prepared via volumetric dilution.

### 2.7 Cell Viability and IC50 Determination

THP‐1‐M and MH‐S cells were seeded at 6,000 cells per well in 96‐well plates and allowed to adhere overnight. The cells were then exposed to 0%–20% CSE for 72 h. Cell viability was measured via the Cell Counting Kit‐8 (CCK‐8) assay (Beyotime, # C0039). After the working solution was added according to the manufacturer's instructions, the plates were incubated at 37°C for 1–2 h, and the absorbance at 450 nm was read. Blank wells were used for background subtraction. The signals were normalized to those of the vehicle controls. Dose‒response curves were fitted in GraphPad Prism (version 9.0) via a four‐parameter logistic non‐linear model to calculate the IC50.

### 2.8 Immunofluorescence For DNA Damage (γ‐H2AX)

The cells were seeded onto sterile coverslips in 24‑well plates. After treatment, the cells were fixed with 4% paraformaldehyde for 15 min at room temperature and washed with PBS. The cells were permeabilized with 0.2% Triton X‑100 for 10 min and blocked with 5% bovine serum albumin (BSA) for 30–60 min. DNA damage was detected via a γ‑H2AX immunofluorescence kit (Beyotime, #C2036S) according to the manufacturer's instructions. Briefly, the cells were incubated with a primary anti‑γ‑H2AX antibody (1:500) overnight at 4°C, washed with PBS, and then incubated with a fluorescent secondary antibody (1:500–1:1000) for 1 h at room temperature. Nuclei were counterstained with DAPI, and coverslips were mounted.

### 2.9 Intracellular reactive oxygen species (ROS) Measurement

Dihydroethidium (DHE) was used as a superoxide‐sensitive probe (Aladdin, #R353922). The cells were incubated with 5–10 µm DHE for 30 min at 37°C in the dark and then gently washed with PBS two to three times. All conditions were processed in parallel and acquired concurrently to minimize intra‐assay variability. Fluorescence microscopy (Olympus, #BX53) was used for acquisition, and the results are reported as the relative fluorescence intensity or mean fluorescence intensity (MFI).

### 2.10 Cellular Senescence Assay

SA‐β‐gal staining was performed with a commercial kit according to the manufacturer's instructions (Beyotime, #RG0039). To avoid artifacts from overconfluence or contact inhibition, the plating density was controlled at 30%–50% confluence. After treatment, the cells were fixed and stained per the protocol and then incubated for 4–16 h at 37°C in a CO2‐free incubator until the SA‐β‐gal–positive cells formed stable blue‐green precipitates. For each well, ≥5 random bright‐field images were acquired. At least 500 cells per group were counted in total. SA‐β‐gal positivity was defined by a prespecified threshold referenced to negative controls. Counting was conducted in a blinded manner.

### 2.11 Phagocytic Activity Assay

Dead bacteria were prepared by heat‐killing at 80°C for 30 min. The bacterial pellets were then labeled with PHK26 red fluorescent dye (2 µm) in PBS at room temperature for 5 min, followed by three washes with PBS to remove excess dye. The bacterial concentration was quantified via OD_600_ measurements. For the phagocytosis assay, PHK26‐labeled dead bacteria were added to PMA‐differentiated THP‐1 cells at a multiplicity of infection (MOI) of 10:1 (bacteria: cell) and coincubated at 37°C for 2 h. Following incubation, the extracellular bacteria were removed by washing three times with cold PBS. The cells were collected by scraping, fixed with 4% paraformaldehyde for 15 min, and analyzed via flow cytometry. Phagocytic activity was quantified as the percentage of PHK26‐positive THP‐1 cells among total THP‐1 cells. The cells incubated at 4°C were used as a negative control to distinguish active phagocytosis from nonspecific bacterial adhesion.

### 2.12 Real‐Time Quantitative‐ PCR (RT‐qPCR)

Total RNA was extracted with TRIzol (Invitrogen, #15596026CN) or an equivalent reagent following the manufacturer's protocol. RNA purity was assessed via a NanoDrop spectrophotometer (Thermo Scientific, A260/280 = 1.8–2.1, A260/230 > 1.8). Integrity was evaluated by agarose gel electrophoresis or a Bioanalyzer (RIN ≥ 7 or clear 28S/18S bands). Reverse transcription was performed via a mixed priming strategy with random hexamers and oligo(dT), with 500 ng–1 µg of RNA input in a 20 µL reaction. Primers were designed to span introns for target genes (e.g., GEM, CDKN1A, and CDKN2A) and the reference gene ACTB, yielding amplicons of 80–200 bp. The primer sequences are listed in Table S1. SYBR Green Master Mix (Thermo Scientific, #A25742) was used. The typical cycling conditions were 95°C for 30 s, followed by 40 cycles of 95°C for 5–10 s and 60°C for 30 s. Each condition included at least three biological replicates. Relative expression was calculated via the 2^‐ΔΔCt method with ACTB as the internal control and the vehicle or baseline sample as the calibrator.

### 2.13 Western Blot

The cells were lysed on ice for 30 min in RIPA buffer (Beyotime, #P0013C) supplemented with protease and phosphatase inhibitors (Beyotime, #SG2000) with intermittent vortexing. The lysates were clarified by centrifugation at 12 000 × g for 15 min at 4°C, and the supernatants were collected. The protein concentration was measured via a bicinchoninic acid (BCA) assay (Beyotime, #P0011), and equal amounts were loaded. Proteins were resolved on 8%–12% SDS‐PAGE gels according to their molecular weight and transferred to PVDF membranes (Millipore, IPVH00010, 0.45 µm). The membranes were blocked with 5% BSA in Phosphate Buffered Saline with Tween 20 (PBST) for 60 min at room temperature. Primary antibodies against GEM (ThermoFisher, #PA5‐55188), p16 (Proteintech, #10883‐1‐AP), p21 (Proteintech, #10335‐1‐AP), SIRT3 (Proteintech, #10099‐1‐AP), SOD2 (Proteintech, #24127‐1‐AP) and ACTB (Proteintech, #66009‐1‐Ig) were incubated overnight at 4°C (1:500–1:2,000 per the vendor recommendations). HRP–conjugated secondary antibodies (Proteintech, #RGAR001 and RGAM001) were applied for 1 h at room temperature (1:10,000). The signals were detected via enhanced chemiluminescence (ECL) (Solarbio, # PE0010). Densitometry was performed in ImageJ: target proteins were normalized to ACTB.

### 2.14 Enzyme linked immunosorbent assay (ELISA) For Cytokines

The culture supernatants were collected after treatment and centrifuged at 1000 × g for 10 min at 4°C to remove cells and debris, followed by shaking at 12,000 rpm for 10 min for further clarification. The supernatants were aliquoted into low‐binding tubes and stored at −80°C, avoiding freeze–thaw cycles. Commercial ELISA kits for human MMP1 (Abcam, #ab215083) and IL‐1β (Beyotime, # PI305) were used per the manufacturers’ protocols. Pilot tests determine appropriate dilution factors to ensure that readings are within the linear range of the standard curve. The plates were read at 450 nm with background correction at 570/630 nm. Standard curves were fitted with a four‐parameter logistic (4‐PL) model.

### 2.15 Gene Overexpression and Knockdown

For overexpression, the open reading frame of human or mouse GEM was cloned and inserted into the pCDH‐CMV‐MCS‐EF1‐copGFP‐T2A‐Puro (Addgene, #72263) expression vector. For knockdown, sense and antisense oligonucleotides encoding shRNAs targeting GEM were designed, synthesized, annealed, and ligated into the pGreenPuro plasmid (YouBio, #VT8180). MH‐S cells were transfected via lipofection and switched to complete medium 6 h later. THP‐1 cells were transfected in suspension and then differentiated with PMA. Forty‐eight hours post‐transfection, the knockdown or overexpression of these genes was validated via qPCR and Western blotting. An empty vector or non‐targeting shRNA served as controls.

### 2.16 Pharmacologic Inhibition and Treatment

Pharmacologic activation of SIRT3 was performed with SIRT3 activator 1 (MedChemExpress, MCE, # HY‐163987). A 10 mm stock solution was prepared in DMSO. The final working concentration was 10 µM (vehicle ≤ 0.1% v/v). The cells were pretreated for 1 h before being subjected to the relevant stimuli (e.g., GEM overexpression or CSE exposure) and maintained at the same concentration throughout. For experiments lasting ≥ 24 h, fresh drug‑containing medium was replaced daily. Pilot tests across 1–10 µm identified 10 µM as the minimal effective concentration without detectable cytotoxicity. On‑target interaction was verified by Western blotting as a restoration of SIRT3 and SOD2 protein levels and by flow cytometry as a reduction in mitochondrial ROS, which was consistent with increased SIRT3 activity.

### 2.17 Cell Cycle Analysis

After treatment, the cells were fixed overnight at −20°C in prechilled 70% ethanol. Following PBS washes, the samples were incubated with RNase A (Beyotime, #ST576, 100 µg/mL, 37°C, 30 min) and stained with propidium iodide (PI, Beyotime, #ST511, 50 µg/mL, protected from light, 30 min). The signals were acquired on a flow cytometer at an excitation wavelength of 488 nm in the red channel. For each sample, ≥20 000 single events were collected. Debris and doublets were excluded by FSC/SSC and pulse geometry. The cell cycle phases (G0/G1, S, and G2/M) were estimated in FlowJo via the Watson Pragmatic model.

### 2.18 Mitochondrial Membrane Potential Measurement via the JC‐1 Assay

The mitochondrial membrane potential (ΔΨm) was assessed via the JC‐1 assay (Invitrogen, Cat. #T3168). THP‐1 m and MH‐S cells were exposed to CSE with or without SIRT3 activator 1 treatment for 24 h. The cells were then incubated with 1 µm JC‐1 dye for 30 min at 37°C. After being washed with PBS, the cells were resuspended in PBS containing 1% FBS. Flow cytometry was performed using a BD FACSCalibur flow cytometer, and the red/green fluorescence ratio was analyzed to assess the mitochondrial membrane potential. The data were analyzed via FlowJo v10 software, with the fluorescence intensity normalized to that of the control conditions.

### 2.19 Mitochondrial Quality Assessment via MitoTracker Staining

Mitochondrial quality was measured by MitoTracker Green FM staining (Invitrogen, Cat. #M7514). THP‐1 m and MH‐S cells were exposed to CSE for 24 h, followed by treatment with a SIRT3 activator 1. The cells were stained with 200 nm MitoTracker Green for 30 min at 37°C, washed with PBS, and analyzed by flow cytometry. MitoTracker fluorescence intensity, which is indicative of mitochondrial mass, was quantified via FlowJo v10 software. The results are expressed as the relative mitochondrial abundance compared with that in control cells.

### 2.20 Cell Proliferation and Viability Assays

For the CCK‑8 assays, the cells were seeded at an optimized density in 96‑well plates. At 72 h, CCK‑8 working solution (Beyotime, # C0046, ≈10% of the culture volume) was added, and the plates were incubated at 37°C for 1–2 h. The OD450 was recorded (optional reference 650 nm). Blank subtraction was applied. The signals were normalized to those of vehicle controls at the same timepoint to compute relative viability and to plot growth curves. DNA synthesis was assessed by 5‐Ethynyl‐2'‐deoxyuridine (EdU) incorporation (Beyotime, #C0071S). EdU (10 µm) was added 2 h before the endpoint. Fixation, permeabilization, and the click reaction (fluorescent labeling) followed the kit protocol. EdU positivity was quantified by random‑field fluorescence microscopy or by flow cytometry.

### 2.21 In Vitro RNA Sequencing and Differential Expression Analysis

THP‐1‐M cells were exposed to 5% CSE for 72 h. Vehicle‑treated cells harvested in parallel served as controls. Total RNA was extracted with TRIzol and quality‑checked via a NanoDrop spectrophotometer and agarose gel electrophoresis. Poly(A)‑selected mRNA libraries were prepared and sequenced on an Illumina NovaSeq platform with paired‑end reads. The raw data quality was assessed with FastQC/MultiQC. Adapters and low‑quality bases were trimmed with Trimmomatic. The reads were aligned to GRCh38 with STAR (v2.7). Gene‑level counts were generated by featureCounts via Gencode annotations. Low‑abundance genes were filtered (e.g., Counts Per Million (CPM) > 1 in ≥50% of the samples). Differential expression analysis was performed with DESeq2. The significance thresholds were |log2FC| ≥ 1 and False Discovery Rate (FDR) p < 0.05.

### 2.22 Transcription Factor Prediction and Validation

Transcription factor (TF) prediction: The TFTF tool (https://jingle.shinyapps.io/TF_Target_Finder/) was used to predict upstream TFs regulating GEM [20]. Putative TF binding sites in the GEM promoter were further assessed via JASPAR 2024. Dual‑luciferase reporter assay: The GEM promoter region was cloned and inserted into a pGL3 reporter. The macrophages were co‑transfected with the TF overexpression plasmid and the Renilla control plasmid pRL. Luciferase activities were measured with a dual‑luciferase system. Transcriptional activation was evaluated by the firefly/Renilla ratio.

### 2.23 M^6^A Regulation Prediction and Validation

The SRAMP algorithm was used to predict the distribution of m^6^A sites in the mature GEM RNA sequence [21]. Primers targeting high‐confidence regions were designed for Methylated RNA Immunoprecipitation (MeRIP) analysis. m^6^A modification in macrophages was examined with a Magna MeRIP m^6^A kit (Millipore, #17–10499) according to the manufacturer's instructions. Briefly, 150 µg of total RNA was extracted and randomly fragmented into ∼100 bp fragments. RNA was immunoprecipitated with beads coated with anti‑m^6^A or anti‑mouse IgG. m^6^A‑enriched fragments were eluted and analyzed by RT‑qPCR. The enrichment data were normalized to the input.

### 2.24 RNA Stability Assay

The RNA decay assays followed published methods [22]. Macrophages transfected with pIGF2BP2 or an empty vector were plated in 6‑well plates. Actinomycin D (Fdbio Science, #FD8226) was added to a final concentration of 10 µg/mL. The cells were collected at 0, 1, 2, 3, 4, and 5 h. RNA was extracted and quantified via RT‑qPCR. RPN1 mRNA was measured with the same primers. Ct values were normalized to t = 0 to obtain the ΔCt (ΔCt = mean Ct at each time point − mean Ct at t = 0). The relative abundance was calculated as the 2^‐ΔCt value.

### 2.25 Gene Expression Omnibus (GEO) Data Analysis

Raw expression matrices and clinical annotations for GSE130928 [23] were downloaded from GEO. Differentially expressed genes between smokers and nonsmokers were identified via the limma package, applying the same significance thresholds as those described above. Gene–gene correlations were assessed by Pearson's correlation, reporting the correlation coefficient (r) and two‐tailed *p* values.

Significantly differentially expressed genes served as input, with the background defined as all detectable genes passing expression filters. Overrepresentation analyses for the Gene Ontology (GO) biological process and Kyoto Encyclopedia of Genes and Genomes (KEGG) pathway analyses were performed via the DAVID online tool [24]. For single‐gene enrichment centered on GEMs, all genes within macrophages in GSE130928 were ranked by their Pearson correlation with GEM expression. Gene set enrichment analysis (GSEA) was then conducted to identify enriched pathways. Key biological processes and signaling pathways were visualized with lollipop plots.

### 2.26 Mouse Cigarette Smoking Model Construction

Male C57BL/6J mice (8 weeks old, 20–25 g; n = 6 per group) were obtained from Beijing Vital River Laboratory Animal Technology Co., Ltd. and housed under Specific Pathogen Free (SPF) conditions at Soochow University (ethics approval no. 202501A014). The mice were randomly assigned to a cigarette smoke (CS) group or a sham control group.

CS‑exposed mice were placed in a 20 × 30 × 15 cm chamber and exposed to mainstream smoke from Hong Shuangxi (Red Double Happiness) cigarettes (11 mg tar, 1.1 mg nicotine, 13 mg CO per cigarette) or six cigarettes/day (three in the morning and three in the afternoon; 5 min per cigarette) for 28 consecutive days. This regimen was designed to establish a controlled, repeated subacute smoke exposure model rather than to replicate lifelong human smoking. Because mice and humans differ substantially in body weight, respiratory rate, airway anatomy, and smoke inhalation behavior, a direct cigarette‐per‐day equivalence to human smoking is not appropriate; the findings should be interpreted as reflecting smoke‐induced biological responses under standardized experimental conditions. Sham mice were exposed to room air. Twenty‑four hours after the final exposure, the mice were deeply anesthetized and euthanized by cervical dislocation, and the lungs were collected for bronchoalveolar lavage and subsequent analyses. Detailed procedures for the smoke exposure system and model construction are provided in Supporting Information S1.

### 2.27 Superoxide dismutase (SOD) Enzyme Activity Assay

SOD activity in the bronchoalveolar lavage fluid (BALF) of the mice was measured via an enzyme‐linked immunosorbent assay (ELISA; Macklin, #M709946). BALF was first collected by washing the alveoli with PBS, which was instilled, and then aspirated via a bronchoalveolar lavage procedure. SOD activity was determined via the colorimetric method at a wavelength of 450 nm. The concentration of SOD in each sample was calculated on the basis of a standard curve. The experimental procedure involved sample preparation, incubation, washing, and color development, ensuring that all steps followed the manufacturer's instructions precisely.

### 2.28 The malondialdehyde (MDA) Content Assay

MDA content in the BALF was measured via ELISA. After the BALF samples were collected, they were washed with PBS and analyzed according to the ELISA kit instructions (Macklin, #H713370). The MDA concentration was calculated by measuring the sample's absorbance at a wavelength of 532 nm. This step ensured that the absorbance data from each sample were accurate and reliable for reflecting the level of oxidative stress.

### 2.29 Immunohistochemical Analysis

Immunohistochemical staining was performed with antibodies against P16, P21, and P53 to evaluate the expression of cell cycle‐related proteins in the lung tissue of the smoking group. Lung tissue from the mice was excised, fixed, dehydrated, and embedded with a thickness of 5 µm. The tissue sections were incubated overnight with primary antibodies against p16 (Proteintech, #10883‐1‐AP), p21 (Proteintech, #10335‐1‐AP), and P53 (Proteintech, #10442‐1‐AP), followed by incubation with an horseradish peroxidase (HRP)‐conjugated secondary antibody. After staining, the samples were examined under a microscope, and images were captured. Image analysis software was used to quantitatively analyze the area of positive cells in each section, and the percentage of positive cells was calculated to assess the effect of smoking on cell cycle protein expression.

### 2.30 Bronchoalveolar Lavage Fluid Collection and Macrophage Isolation

Bronchoalveolar lavage fluid was collected via bronchoalveolar lavage and placed in centrifuge tubes. After centrifugation, the supernatant was discarded. The macrophages were then isolated via the adherent method. The samples were allowed to stand for 30 min, after which nonadherent cells were discarded, and the adherent macrophages were counted. The number of pulmonary alveolar macrophages in each group was recorded and compared with that in the Sham group to analyze the impact of smoking on pulmonary immune responses.

### 2.31 Statistical Analysis

Unless swise specified, all in vitro experiments were performed with three independent biological replicates per group (each based on ≥3 technical replicates that were averaged before statistical testing), and all in vivo mouse experiments were conducted with six biological replicates per group. Continuous variables were examined for normality using the Shapiro–Wilk test and for homogeneity of variance using Levene's test. Data are presented as mean ± standard deviation (SD) unless indicated otherwise. For comparisons between two groups, a two‑sided unpaired Student's t test was used when the assumptions of normality and equal variance were satisfied. For comparisons among more than two groups, one‑way analysis of variance (one‑way ANOVA) followed by Tukey's multiple‑comparison post hoc test was applied under the same assumptions. Pearson's correlation analysis was used to assess linear associations between continuous variables. A two‑sided alpha level of 0.05 was considered statistically significant (*p* < 0.05). All statistical analyses and graphing were performed using GraphPad Prism (version 9.0) and R (version 4.4.2).

## 3 Results

### 3.1 Alveolar Macrophages in Smokers’ BALF Exhibit a Senescent Phenotype

**FIGURE 1:**
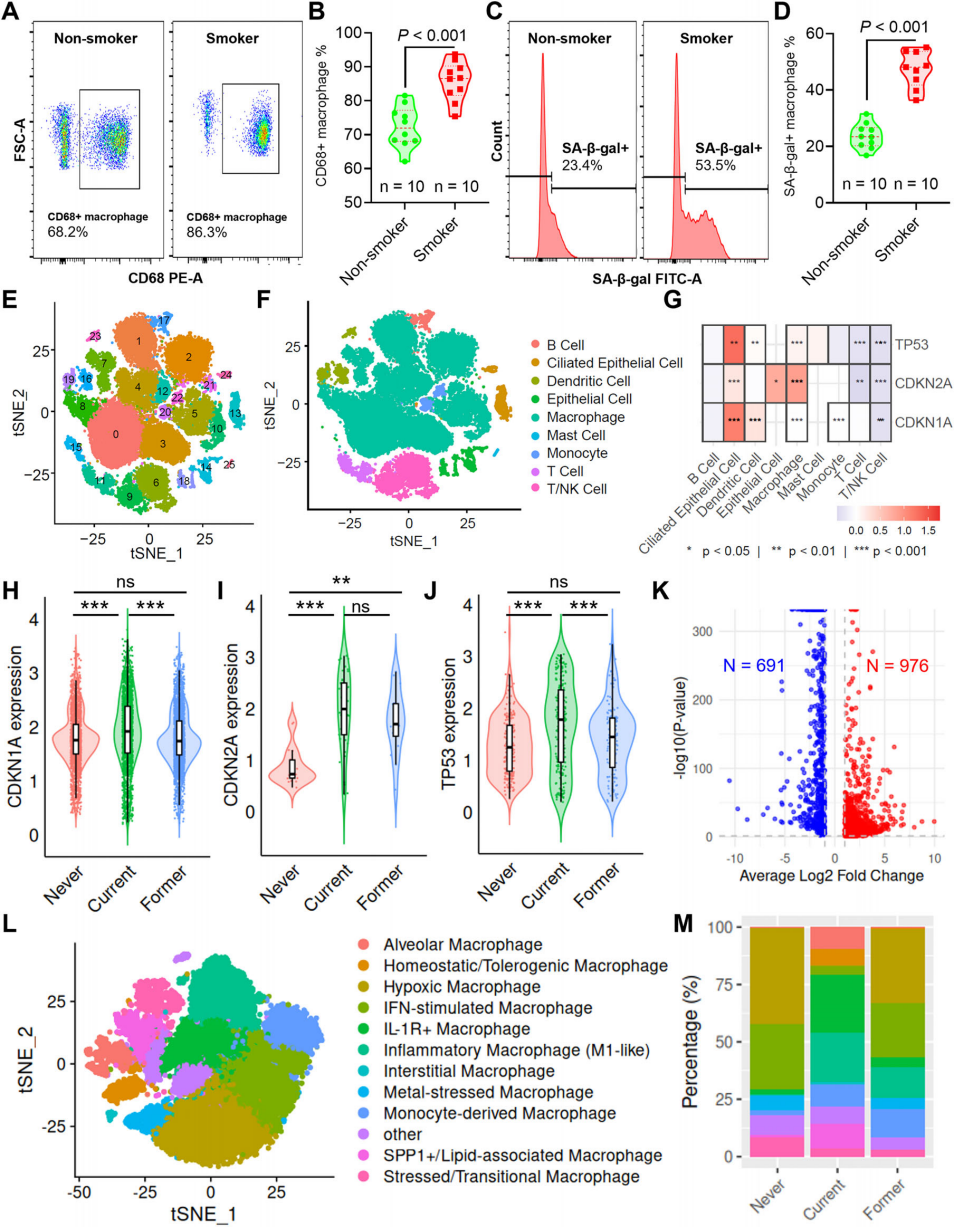
Flow cytometry and scRNA‑seq analysis of BALF cells from clinical samples. (A) Representative flow cytometry plots showing CD68^+^ macrophages in the BALF of nonsmokers and smokers. (B) Quantification of CD68^+^ macrophage proportions in BALF from nonsmokers (n = 10) and smokers (n = 10). (C) Representative flow cytometry plots showing SA‐β‐gal–positive macrophages in CD68^+^ macrophages of nonsmokers and smokers. (D) Quantification of SA‑β‑gal–positive macrophages in BALF from nonsmokers (n = 10) and smokers (n = 10). (E‐F) tSNE plots of BALF cells from never (n = 3), former (n = 2), and current (n = 3) smokers, showing clustering and major cell‑type annotation. (G) Heatmap of fold‑change differences in CDKN1A, CDKN2A, and TP53 across major cell types in current vs. never smokers. Expression of CDKN1A (H), CDKN2A (I), and TP53 (J) in alveolar macrophages according to smoking status. (K) Volcano plot showing DEGs between macrophages from smokers and never‐smokers in the BALF scRNA‐seq dataset. (L) tSNE visualization of macrophage subpopulation annotations derived from re‐clustering of macrophage cells isolated from the primary clustering analysis. (M) A stacked bar chart showing the distribution of macrophage subpopulations obtained from further sub‐clustering. DEGs, differentially expressed genes. SA‑β‑gal, senescence‑associated β‑galactosidase; UMAP, uniform manifold approximation and projection; BALF, bronchoalveolar lavage fluid. ^*^ *p* < 0.05; ^**^ *p* < 0.01; ^***^ *p* < 0.001.

We profiled BALF cells from individuals with different smoking histories using flow cytometry and scRNA‐seq. Flow cytometry showed a higher proportion of CD68^+^ macrophages in smokers than in nonsmokers (Figure 1A,B). Within the macrophage compartment, smokers had a significantly greater fraction of SA‐β‐gal–positive cells (Figure 1C,D), indicating that smoking is associated with enhanced macrophage senescence.

To chart the BALF cellular landscape, we performed scRNA‐seq on BALF cells from never (n = 3), former (n = 2), and current (n = 3) smokers. t‐SNE analysis yielded stable and reproducible clusters that were annotated into major cell types based on canonical marker genes (Figure 1E,F; Figure S1A). A heatmap of senescence‐related genes across major cell types (Figure 1G) showed that CDKN1A (encoding p21), CDKN2A (encoding p16), and TP53 (encoding p53) were markedly upregulated in multiple cell subsets from current smokers compared with never smokers, with particularly prominent increases in macrophages. Consistently, at the single‐cell level, expression of CDKN1A, CDKN2A, and TP53 in alveolar macrophages was significantly higher in current smokers than in never and former smokers (Figure 1H–J), indicating that smoking preferentially drives a senescence‐associated transcriptional program in the macrophage compartment. We next examined the SASP at single‐cell resolution. Analysis of cytokine and SASP‐encoding genes across major cell types (Figure S1B) revealed broadly increased expression of multiple pro‐inflammatory mediators in BALF cells from current smokers compared with never smokers. This upregulation was observed not only in macrophages but also in T cells and other immune and structural cell populations, suggesting that smoking is associated with a SASP‐like, cytokine‐rich microenvironment in the airways.

Differential expression analysis of macrophages from smokers versus never‐smokers identified 1,667 differentially expressed genes (DEGs), including 691 downregulated genes and 976 upregulated genes in smokers (Figure 1K). Gene Ontology and pathway enrichment analyses showed that these DEGs were significantly enriched in biological processes such as cell adhesion, immune system process, inflammatory response, and response to cytokine, and in signaling pathways involving TNF, NF‐κB, and FoxO, as well as pathways related to cellular senescence (Figure S1C,D). These results indicate that smoking induces a senescence‐linked, pro‐inflammatory transcriptional program in alveolar macrophages. To further resolve macrophage heterogeneity, we reclustered BALF macrophages and identified distinct macrophage subpopulations (Figure 1L; Figure S2A–C). Analysis of subset composition revealed that smokers exhibited an increased proportion of inflammation‐associated macrophages, including IL‐1R^+^ and M1‐like macrophages, as well as SPP1^+^ (SPP^+^) macrophages, compared with never smokers (Figure 1M). In contrast, the macrophage subset distribution in former smokers largely resembled that of never smokers, suggesting partial reversibility of smoking‐induced macrophage reprogramming after smoking cessation.

Together, these data demonstrate that smoking is associated with a senescent and pro‐inflammatory transcriptional state in alveolar macrophages and other BALF cell populations, accompanied by remodeling of macrophage subset composition in the lung.

### 3.2 CSE Exposure Induces Macrophage Senescence

**FIGURE 2:**
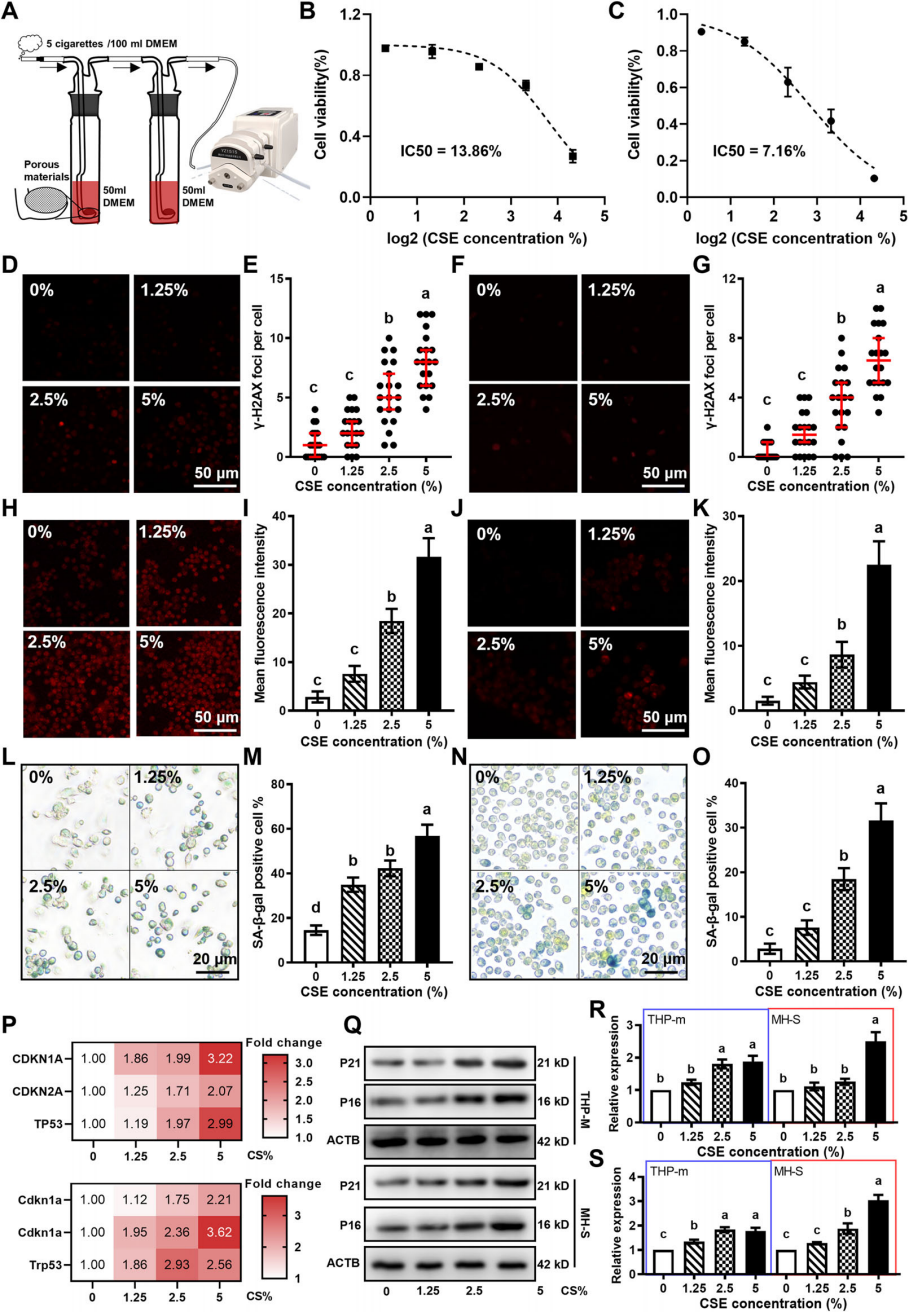
In vitro CSE treatment induces macrophage senescence. (A) Schematic diagram of the cigarette smoke extract (CSE) preparation apparatus. (B–C) Dose–response curves of cell viability in PMA‑differentiated THP‑1 macrophages (THP‐1‑M, B) and mouse alveolar macrophages (MH‑S, C) after increasing concentrations of CSE. (D–G) γ‑H2AX immunofluorescence showing representative images and quantification of nuclear DNA damage in THP‐1‑M (D–E) and MH‑S (F–G) cells following CSE exposure. (H–K) DHE staining showing representative images and quantification of intracellular ROS levels in THP‐1‑M (H–I) and MH‑S (J–K) cells after CSE treatment. (L–O) Representative images and quantification of SA‑β‑gal activity in THP‐1‑M (L–M) and MH‑S (N–O) cells following CSE exposure. (P) Heatmap summarizing the changes in the expression of three senescence‑related genes in THP‐1‐M and MH‐S cells after CSE treatment via a qPCR‐based approach. (Q) Western blot analysis of P16 and P21 protein expression in THP‐1‐M and MH‐S cells after CSE treatment, with densitometric quantification. Original blots can be found in Supporting Information S2. All the cell culture experiments were performed with three independent biological replicates. Groups that do not share a letter differ significantly (p < 0.05), whereas groups sharing a letter are not significantly different. CSE, cigarette smoke extract; SA‑β‑gal, senescence‑associated β‑galactosidase; ROS, reactive oxygen species.

To model the injurious and prosenescent effects of CSE in vitro, we standardized the CSE preparation (Figure 2A). THP‑1–derived macrophages (THP‐1‐M) and the mouse alveolar macrophage line MH‑S were treated with graded CSE concentrations for 72 h to generate dose‒response curves. CSE suppressed viability in a dose‐dependent manner in both lines, with IC50 values of 13.86% (THP‐1‐M) and 7.16% (MH‐S) (Figure 2B,C). We selected three lower‐toxicity doses—1.25%, 2.5%, and 5%—to establish a CSE‐induced macrophage senescence model.

γ‑H2AX immunofluorescence revealed a dose‐dependent increase in the number of nuclear γ‑H2AX signals and foci in both cell types (Figure 2D–G). DHE staining revealed a corresponding dose‐dependent increase in the level of intracellular ROS (Figure 2H–K). Flow cytometry revealed increased apoptosis in THP‐1‑M cells with increasing CSE doses (Figure S3A,B). Phagocytosis, measured via fluorescently labeled bacteria in THP‐1‑M cells, decreased in a dose‐dependent manner (Figure S3C,D). Senescence features increased in parallel: SA‑β‑gal positivity increased with increasing CSE concentration (Figure 2L–O). Consistent with these phenotypes, qPCR revealed dose‐dependent induction of CDKN1A and CDKN2A (Figure 2P), and Western blotting confirmed corresponding increases in p21 and p16 protein levels (Figure 2Q). Together, these data establish CSE as an injurious and prosenescent stimulus for macrophages and provide a robust in vitro platform for mechanistic and intervention studies.

### 3.3 Identification Of GEM as a Key Gene in CSE‑Induced Macrophage Senescence

To identify and validate drivers of CSE‑induced macrophage senescence, we profiled the transcriptomic response of THP‐1‐M cells after 24 h of exposure to 5% CSE. Differential expression analysis revealed marked remodeling, with 3773 upregulated and 2196 downregulated genes (Figure S4A). Notably, many SASP‑encoding genes were upregulated, and the top three genes were MMP1, MMP3, and IL1B (Figure S4B). ELISA confirmed dose‑dependent increases in MMP1 and IL‑1β in THP‐1‑M supernatants following CSE treatment (Figure S4C,D), supporting the induction of senescence. The enrichment results indicated significant overrepresentation of key processes and pathways (Figure S4E,F), including the cellular response to hypoxia, extracellular matrix organization, cell adhesion, the inflammatory response, and cytokine–cytokine receptor interactions.

**FIGURE 3:**
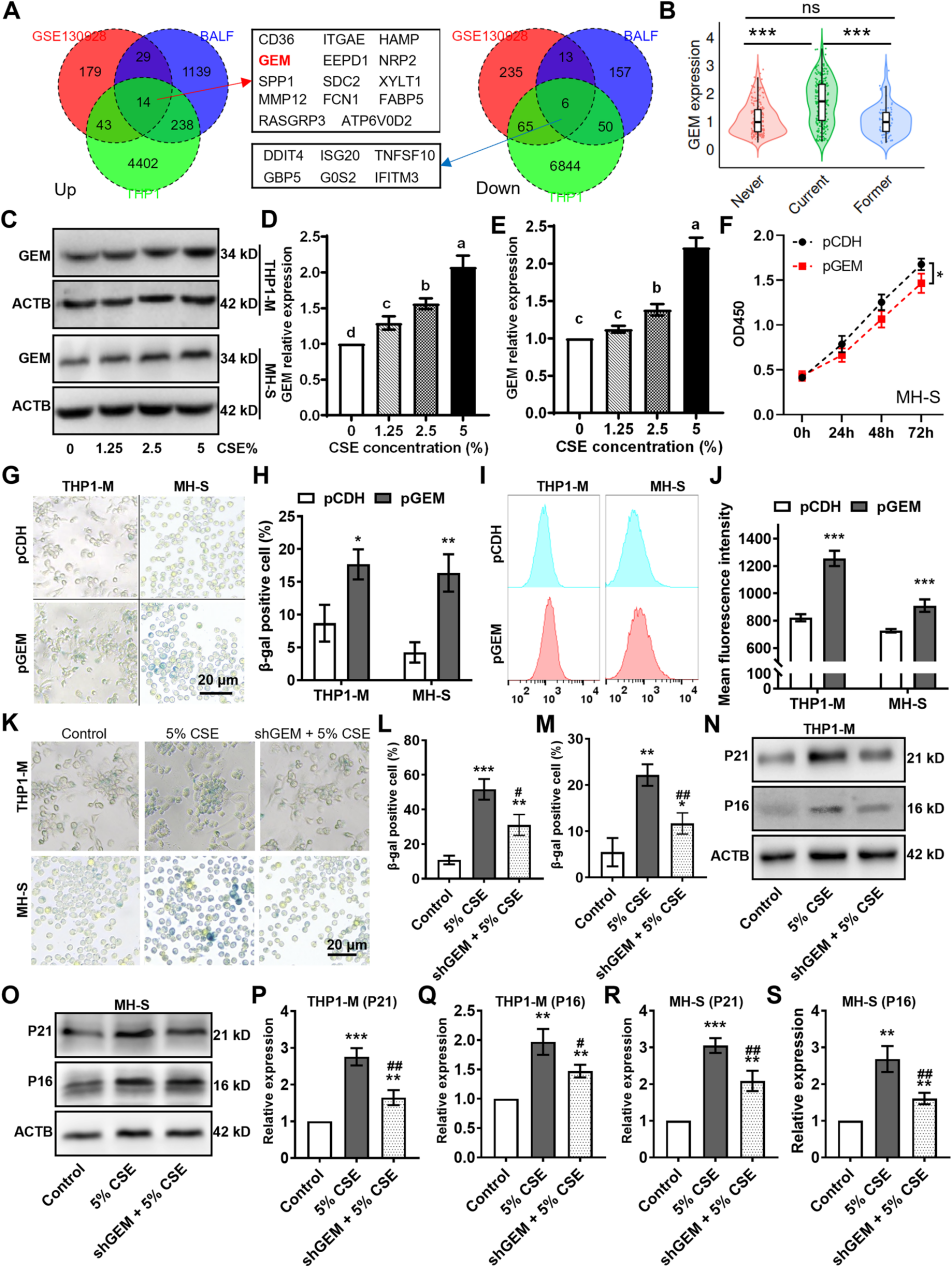
In vitro validation of GEM as a regulator of CSE‑induced macrophage senescence. A three‑way Venn diagram (A) shows the overlap of differentially expressed genes (DEGs) identified from BALF scRNA‑seq of smokers vs. never smokers, GEO‑derived macrophage DEGs (GSE130928), and in vitro CSE‑exposed THP‐1‑M cells, highlighting GEM as a shared candidate. (B) GEM expression in macrophages across smoking histories (never, former, current smokers; 3 donors per group) in the scRNA‑seq dataset (^***^ *p* < 0.001). Western blotting showing GEM protein expression in THP‐1‐M and MH‐S cells treated with increasing concentrations of CSE (C), with densitometric quantification shown in (D,E); groups that do not share a letter differ significantly (*p* < 0.05), whereas groups sharing a letter do not significantly differ. The effect of GEM overexpression on MH‑S cell proliferation was assessed via a CCK‑8 assay (F). Representative SA‑β‑gal‐stained images of THP‐1‑M and MH‑S cells after GEM overexpression (G) and quantification of SA‑β‑gal‐positive cells (H) indicate increased senescence. Flow cytometry showing the impact of GEM overexpression on intracellular ROS levels in both macrophage models (I), with the mean fluorescence intensity (MFI) quantified in (J). Representative SA‑β‑gal images (K) and corresponding quantification (L,M) demonstrate that GEM knockdown protects against CSE‑induced senescence in both models. Western blot analyses of P16 and P21 in CSE‑treated THP‐1‑M (N) and MH‑S (O) cells after GEM knockdown, with densitometric quantification (P‐S). Original blots can be found in Supporting Information S2. All the cell experiments were performed with three independent biological replicates. ^*^, ^**^, and ^***^ indicate *p* < 0.05, *p* < 0.01, and *p* < 0.001, respectively, compared with the control group; # and ## indicate *p* < 0.05 and *p* < 0.01, respectively, compared with the 5% CSE group. CSE, cigarette smoke extract; scRNA, single‑cell RNA sequencing; DEGs, differentially expressed genes; ROS, reactive oxygen species; SA‑β‑gal, senescence‑associated β‑galactosidase.

We next integrated three datasets to identify robust candidates: DEGs in macrophages from smokers versus never smokers identified by clinical BALF scRNA‑seq (this study); DEGs from THP‐1‐M cells exposed to CSE in vitro (this study); and DEGs in macrophages from smokers in the GSE130928 cohort [23]. In GSE130928, 349 genes were upregulated, and 295 were downregulated in smokers’ macrophages (Figure S5A). The enriched genes were associated with the response to IFN‑γ/TNF, cytokine‑mediated signaling, the inflammatory response, Toll‑like receptor signaling, and the JAK–STAT pathway (Figure S5B). The intersection of the three DEG sets defined a candidate gene list (Figure 3A). In GSE130928, many intersecting genes, including GEM, NRP2, ATP6V0D, FABP5, and SDC2, were positively correlated with CDKN1A in macrophages (Figure S6A). GEM showed the strongest correlation with CDKN1A (r = 0.602; Figure S6B). At single‑cell resolution, GEM expression was greater in macrophages from current smokers than in those from never smokers and former smokers (Figure 3B).

**FIGURE 4:**
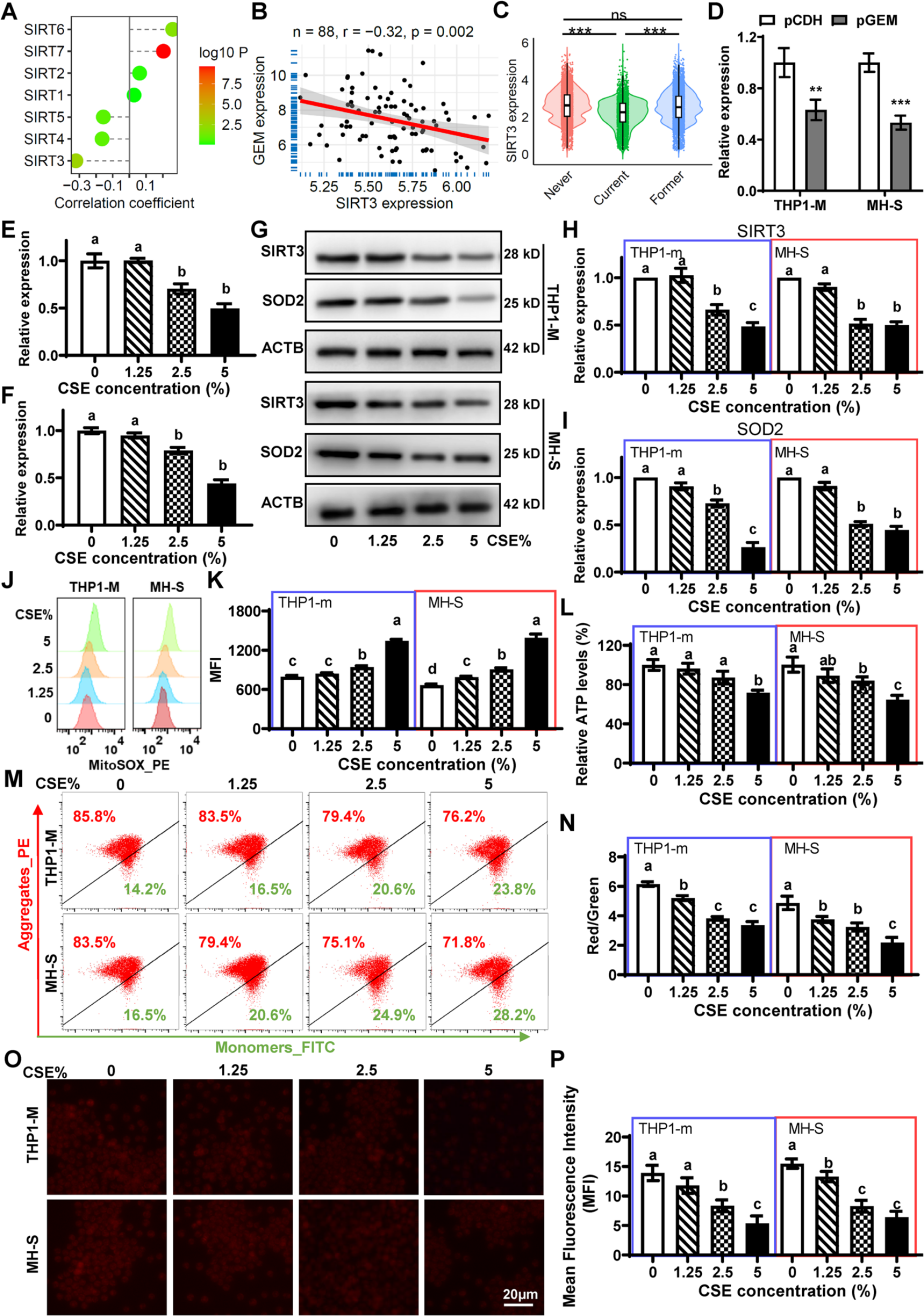
CSE exposure induces mitochondrial damage in macrophages. A lollipop plot (A) shows correlations between GEM and SIRT family gene expression in bulk macrophage transcriptomes from smokers and nonsmokers in GSE130928 (n = 88), with a focused scatter plot (B) illustrating the positive correlation between GEM and SIRT3 expression. (C) SIRT3 expression in BALF macrophages from never, former, and current smokers (3 donors per group; n = 9) in the single‑cell RNA‑seq dataset. (D) qPCR analysis of SIRT3 mRNA levels in macrophages overexpressing GEM. qPCR analysis of SIRT3 expression in THP‐1‑M (E) and MH‑S (F) cells treated with increasing concentrations of CSE. (G–I) Western blot analysis and densitometric quantification of SIRT3 and SOD2 protein expression in macrophages exposed to different concentrations of CSE. Original blots can be found in Supporting Information S2. Flow cytometric measurement of mitochondrial ROS (mitoROS) in CSE‑treated macrophages (J) with quantification of the mean fluorescence intensity (K). (L) Intracellular ATP levels in macrophages exposed to graded CSE doses. Flow cytometric analysis of the mitochondrial membrane potential via JC‑1 staining (M) and quantification of the red/green fluorescence ratio (N) in CSE‑treated macrophages. Flow cytometric measurement of mitochondrial quality via MitoTracker staining (O) and corresponding quantification (P) in CSE‑treated macrophages. All in vitro experiments were performed with three independent biological replicates. qPCR, quantitative polymerase chain reaction; CSE, cigarette smoke extract; ATP, adenosine triphosphate; ROS, reactive oxygen species.

 qPCR and western blotting confirmed the dose‑dependent induction of GEM mRNA and protein expression by CSE in both THP‐1‑M and MH‑S cells (Figure S7C,D and Figure 3C–E). Single‑gene enrichment focused on GEM in GSE130928 linked GEM to cell‑cycle DNA replication, the acute inflammatory response, the response to IFN‑γ/α, and the JAK–STAT signaling pathway (Figure S7E,F). CCK‑8 assays revealed that GEM overexpression suppressed MH‑S proliferation (Figure 3F), whereas GEM knockdown promoted proliferation (Figure S7G). GEM overexpression triggered classical senescence‑like changes in both cell types, including increased SA‑β‑gal positivity (Figure 3G,H). Consistently, flow cytometry revealed elevated intracellular ROS after GEM overexpression (Figure 3I,J). qPCR demonstrated significant induction of CDKN1A and CDKN2A in THP‐1‑M and MH‑S cells (Figure S7H,I). Moreover, GEM knockdown significantly attenuated the increase in SA‑β‑gal positivity induced by 5% CSE in both macrophage models (Figure 4K–M). GEM knockdown partially reversed the CSE‑induced upregulation of CDKN1A (Cdkn1a) and CDKN2A (Cdkn2a) mRNA (Figure S7J,K). Western blot analysis likewise revealed that GEM knockdown blunted the CSE‑induced increase in P16 and P21 protein levels (Figure 3N–S). These results indicate that GEM is required for the full execution of the CSE‑induced macrophage senescence program.

### 3.4 CSE Exposure Induces Mitochondrial Damage in Macrophages

In GSE130928, GEM was correlated with the SIRT family, with the strongest association observed for SIRT3 (r = −0.320, *p* < 0.05; Figure 4A,B). Single‐cell RNA sequencing revealed that macrophage SIRT3 expression varied according to smoking history, with current smokers displaying significantly lower levels than never smokers and former smokers (Figure 4C). Forced expression of GEM reduced SIRT3 mRNA in the two macrophage models (Figure 4D). Consistently, qPCR and Western blot analyses demonstrated that CSE exposure decreased both SIRT3 mRNA and protein levels in a dose‐dependent manner (Figure 4E–I). Flow cytometry revealed that CSE significantly increased mitochondrial ROS (mitoROS) in both macrophage models (Figure 4J,K). Moreover, CSE suppressed intracellular ATP production (Figure 4L), indicating that CSE exposure impaired mitochondrial function. Additionally, JC‐1 staining experiments revealed that CSE treatment significantly reduced the mitochondrial membrane potential in macrophages, with a more pronounced decrease observed in the high‐concentration group (Figure 4M,N). Furthermore, MitoTracker staining revealed a reduction in mitochondrial abundance in CSE‐treated macrophages (Figure 4O,P). Collectively, these data indicate that CSE exposure induces mitochondrial damage in macrophages.

### 3.5 GEM Engages the SIRT3 Pathway to Promote Macrophage Senescence

**FIGURE 5:**
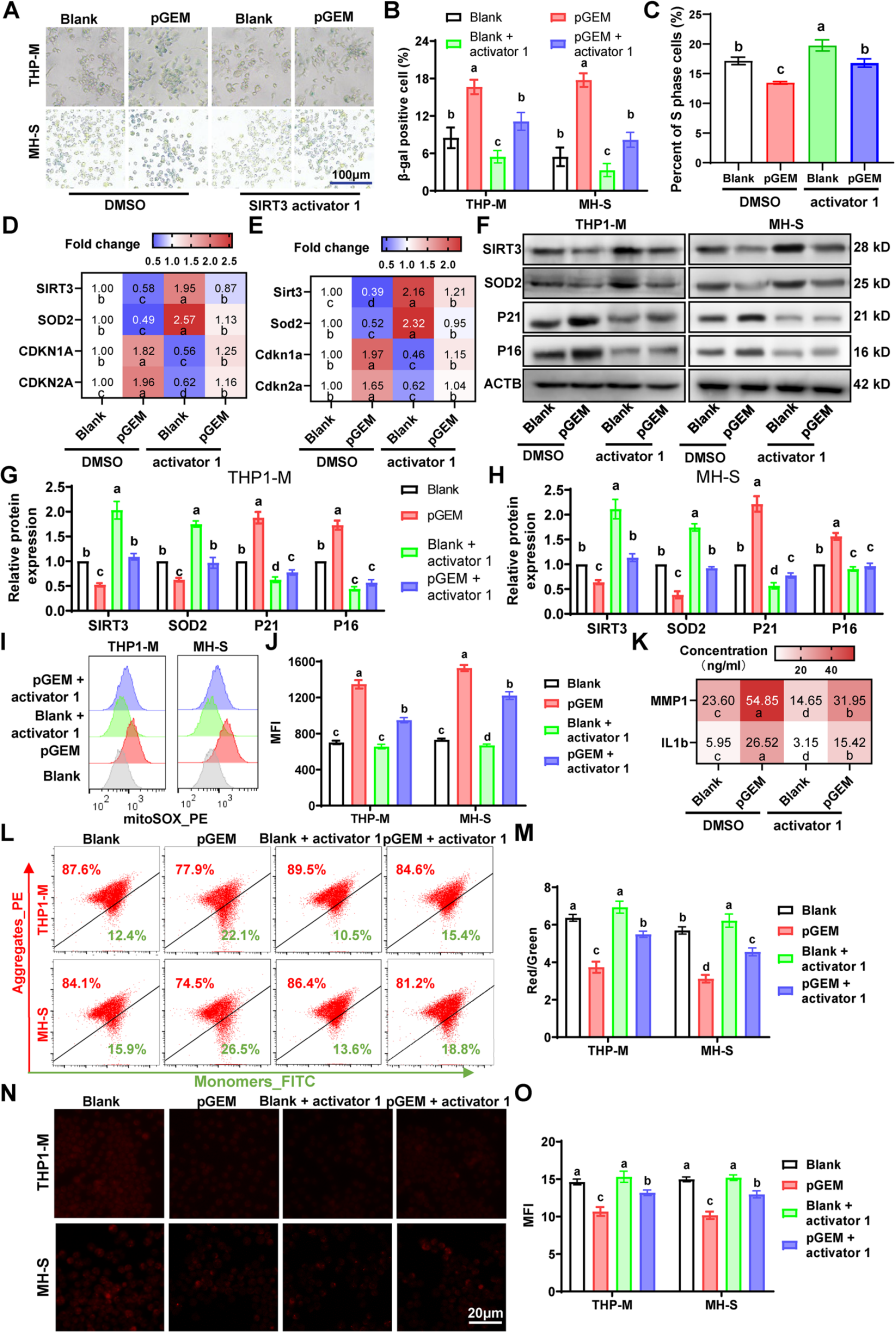
Rescue experiments demonstrated that GEM overexpression induces macrophage senescence via the SIRT3‐mitochondrial pathway. Representative SA‑β‑gal staining images (A) and quantification of SA‑β‑gal–positive cells (B) in THP‐1‐M and MH‑S macrophages with GEM overexpression and/or treatment with the SIRT3 activator 1 illustrate that GEM‑induced senescence is attenuated by SIRT3 activation. (C) Bar plots showing the percentage of S‑phase cells in MH‑S under the same conditions. Heatmaps summarizing the changes in CDKN1A (Cdkn1a), CDKN2A (Cdkn2a), SOD2 (Sod2), and SIRT3 (Sirt3) expression in THP1‑M (D) and MH‑S (E) cells following GEM overexpression and/or SIRT3 activator 1 treatment. (F–H) Western blot analysis and densitometric quantification of SIRT3, SOD2, P16, and P21 protein levels under these conditions. Original blots can be found in Supporting Information S2. Flow cytometric measurement of mitochondrial ROS (mitoROS) (I) and quantification of the mean fluorescence intensity (J) revealed that SIRT3 activation reduces GEM‑induced oxidative stress. (K) Heatmap of MMP1 and IL‐1B levels in THP‐1‑M culture supernatants, reflecting changes in SASP‑related cytokine secretion. Flow cytometric analysis of the mitochondrial membrane potential via JC‑1 staining in THP‐1‑M and MH‑S (L) cells and quantification of the red/green fluorescence ratio (M), together with MitoTracker‑based assessment of mitochondrial quality (N) and corresponding quantification (O), demonstrated that SIRT3 activation mitigated GEM‑induced mitochondrial dysfunction. All the cell experiments were performed with three independent biological replicates. Groups that do not share a letter differ significantly (*p* < 0.05), whereas groups sharing a letter are not significantly different. SA‑β‑gal, senescence‑associated β‑galactosidase; ROS, reactive oxygen species.

In a rescue experiment, we investigated whether GEM overexpression induces macrophage senescence through the SIRT3‐mitochondrial pathway. Phenotypically, SIRT3 activator 1 significantly attenuated the increased SA‐β‐gal positivity caused by GEM overexpression (Figure 5A,B). In MH‐S cells, GEM overexpression reduced the S‐phase fraction, which was consistent with senescence‐associated growth arrest and reversed by SIRT3 activator 1, indicating that the activation of SIRT3 alleviated senescence (Figure 5C). Gene expression analysis via qPCR in both THP‐1‐M and MH‐S cells revealed that SIRT3 activator 1 mitigated the GEM‐induced downregulation of SIRT3 and SOD2 but also blunted the induction of the senescence markers CDKN1A and CDKN2A (Figure 5D,E). Western blot analysis confirmed that GEM overexpression decreased SIRT3 and SOD2 protein levels while increasing P21 and P16, and these effects were antagonized by SIRT3 activator 1 (Figure 5F–H). Flow cytometry further demonstrated that GEM overexpression significantly elevated mitochondrial ROS (mitoROS) levels, which were reduced to control levels upon treatment with SIRT3 activator 1 (Figure 5I,J). Additionally, GEM overexpression increased the secretion of MMP1 and IL‐1β, both of which were reduced following SIRT3 activator 1 treatment (Figure 5K). Flow cytometric analysis revealed that GEM overexpression significantly decreased the mitochondrial membrane potential in both cell types. However, treatment with the SIRT3 activator 1 partially restored the mitochondrial membrane potential, as evidenced by the increased red/green fluorescence ratio compared with that in the CSE‐treated group (Figure 5L,M). Further MitoTracker staining revealed that SIRT3 activator 1 treatment mitigated the loss of mitochondrial mass induced by GEM overexpression, highlighting the potential of targeting the SIRT3 pathway to restore mitochondrial function. (Figure 5N,O). Collectively, these data indicate that GEM drives macrophage senescence by repressing SIRT3 and disrupting mitochondrial homeostasis.

### 3.6 Validation Of Cigarette Smoke‐Induced Macrophage Senescence in a Mouse Model

**FIGURE 6:**
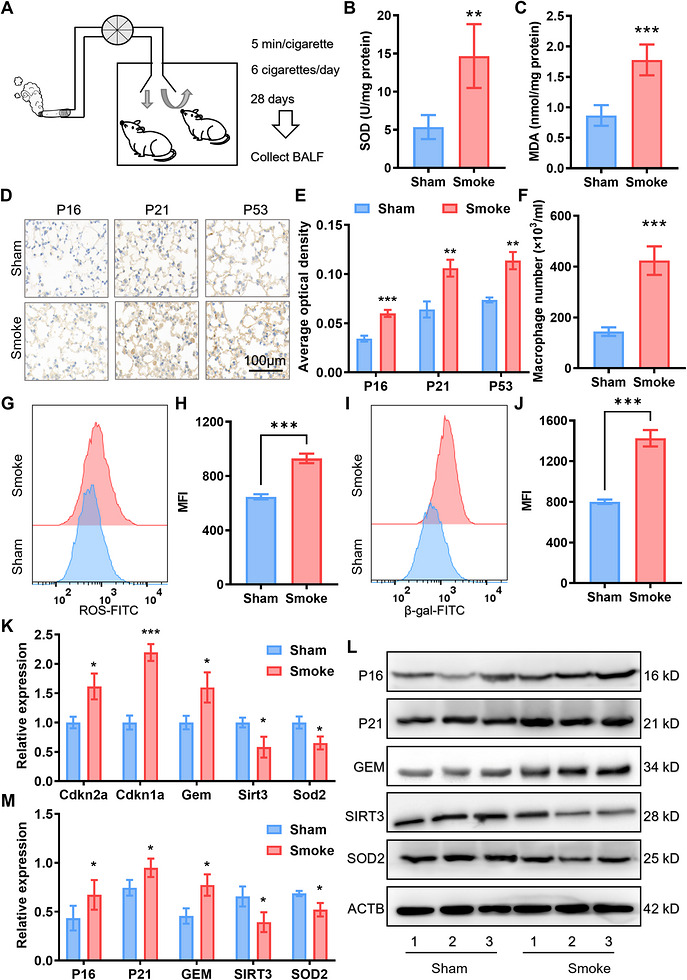
Validation of the role of the GEM/SIRT3 pathway in cigarette smoke–induced macrophage senescence in vivo. A chronic cigarette smoke exposure model was established in male C57BL/6J mice (n = 6 per group), as shown schematically in (A). Oxidative stress in the BALF was assessed via ELISA–based measurement of SOD activity (B) and the MDA content (C). Immunohistochemistry for P16, P21, and P53 in lung tissue (D), with semi‐quantitative analysis (E), revealed increased expression of senescence markers after cigarette smoke exposure. (F) Total macrophage counts in bronchoalveolar lavage fluid confirmed increased macrophage accumulation in the lungs of smoke‐exposed mice. Flow cytometry was used to measure intracellular ROS levels in BALF macrophages (G), with quantitative analysis (H), and β‐galactosidase activity (SA‐β‐gal) in macrophages (I), with corresponding quantification (J), indicating elevated oxidative stress and senescence. (K) qPCR analysis of the expression of genes related to macrophages (including GEM, SIRT3, and senescence–related genes) and Western blot analysis of related proteins (L), with semi‐quantitative densitometric analysis (M), further confirming the activation of the GEM/SIRT3 pathway and senescence signaling in vivo following cigarette smoke exposure. Original blots can be found in Suppoting Information S2. ^*^ *p* < 0.05, ^**^ *p* < 0.01, ^***^ *p* < 0.001 vs. the sham group. BALF, bronchoalveolar lavage fluid; SOD, superoxide dismutase; ELISA, enzyme‐linked immunosorbent assay; ROS, reactive oxygen species; qPCR, quantitative polymerase chain reaction.

We further established a smoking model in mice to evaluate the impact of cigarette smoke on alveolar macrophage senescence (Figure 6A). ELISA analysis of BALF from the mice revealed a significant increase in both SOD activity and MDA levels in the smoking group (Figure 6B,C). Immunohistochemical staining of lung tissue revealed increased expression of the senescence markers P16, P21, and P53 in the lungs of smoking mice (Figure 6D,E). The macrophages were isolated via the adherent method, and their purity was confirmed via flow cytometry. The total number of macrophages in the lung lavage fluid from smoking mice was significantly elevated (Figure 6F). Flow cytometry analysis revealed an increase in ROS levels in macrophages from smoking mice (Figure 6G,H), and β‐galactosidase activity was increased, indicating increased SA‐β‐gal activity (Figure 6I,J). qPCR and Western blot analyses revealed that key senescence‐associated genes, including Cdkn1a, Cdkn2a, Trp53, and Gem, were significantly upregulated in macrophages from the smoking group, whereas Sirt3 and Sod2 were significantly downregulated (Figure 6K,M). Overall, the mouse smoking model strongly supports the role of the GEM/SIRT3 pathway in mediating the effects of cigarette smoke on macrophage senescence.

### 3.7 Upstream Mechanisms Driving GEM Upregulation in CSE‑Induced Macrophage Senescence

**FIGURE 7:**
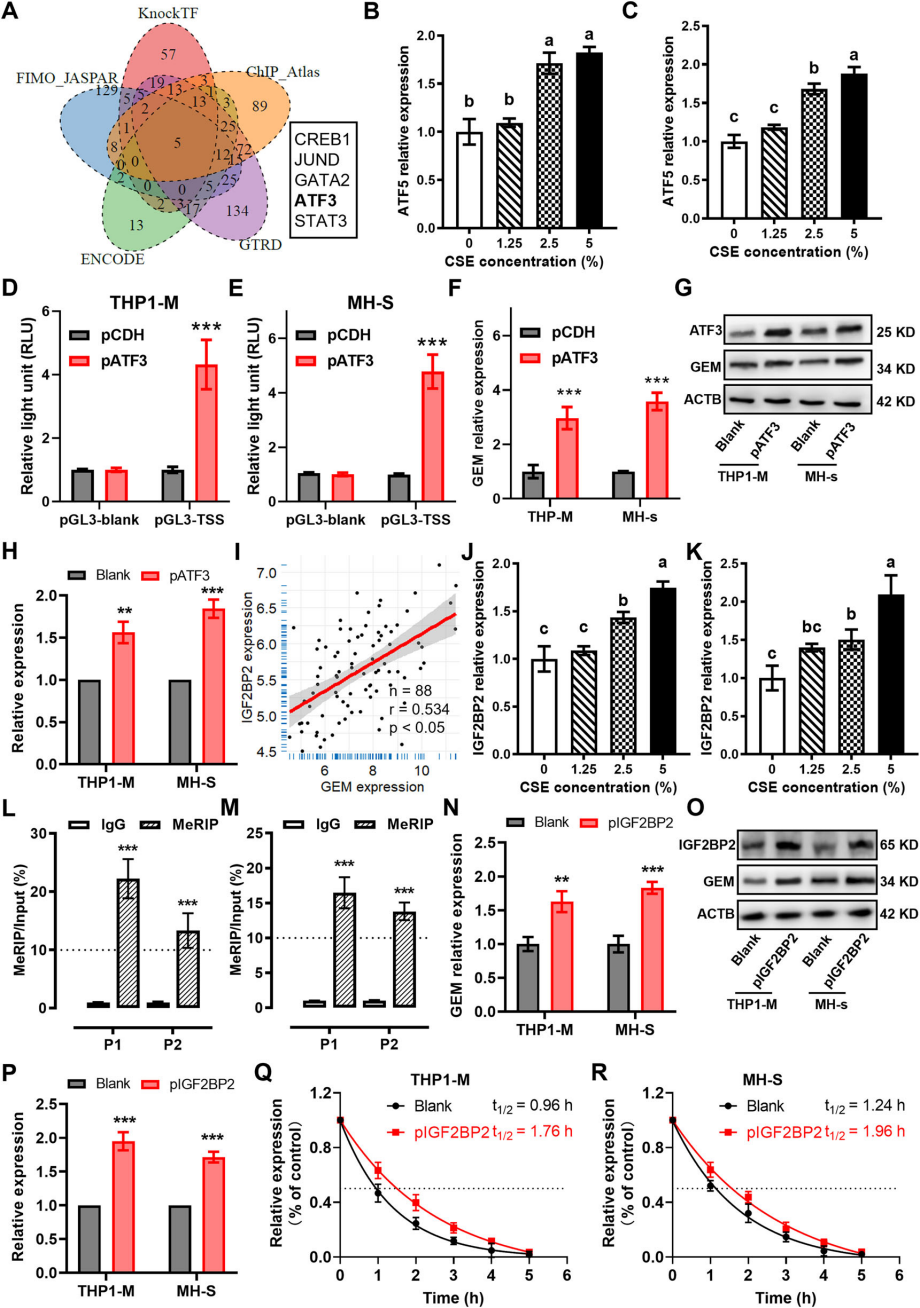
GEM upregulation in CSE‑induced macrophage senescence is regulated by the transcription factor ATF3 and the m^6^A reader IGF2BP2. (A) Venn diagram showing predicted upstream transcription factors for GEM via the TFTF tool. (B,C) qPCR analysis of ATF3 expression in THP‐1‑M and MH‑S cells treated with increasing concentrations of CSE (0%, 2.5%, 5%, or 10%) for 24 h (n = 3 per group). (D,E) Dual‑luciferase assays demonstrating ATF3‑mediated GEM promoter activation in THP1‑M and MH‑S cells (n = 3). (F) qPCR analysis of GEM expression in THP‐1‑M and MH‑S cells after ATF3 overexpression (n = 3). (G,H) Western blot analysis of GEM expression in THP‐1‑M and MH‑S cells after ATF3 overexpression (n = 3). (I) Correlation between GEM and IGF2BP2 in GSE130928. (J,K) qPCR of IGF2BP2 in THP‐1‑M and MH‑S cells treated with increasing concentrations of CSE (n = 3 per group). Groups not sharing letters differ significantly (*p* <0.05). (L‐M) MeRIP assays measuring m^6^A methylation at two GEM sites in CSE‐treated THP‐1‑M and MH‑S cells (n = 3). (N) QPCR analysis of GEM following IGF2BP2 overexpression in both cell lines (n = 3). (O‐P) Western blot analysis of GEM following IGF2BP2 overexpression in both cell lines (n = 3). (Q‐R) RNA stability assays showing IGF2BP2‑dependent GEM mRNA stabilization in THP‐1‑M and MH‑S cells after actinomycin D treatment (n = 3 per time point). Original blots can be found in Supporting Information S2. The data are shown as the means ± SEMs. ^**^, *p* < 0.01; ^***^, *p* < 0.001. CSE, cigarette smoke extract; MeRIP, methylated RNA immunoprecipitation; BALF, bronchoalveolar lavage fluid.

To define how CSE elevates GEM in macrophages, we mapped transcriptional and post‑transcriptional regulation. First, the TFTF tool predicted five candidate upstream transcription factors (TFs) (Figure 7A). In GSE130928, ATF3 and JUND correlated positively with GEM, with ATF3 showing the strongest association (r = 0.796, Figure S8A,B). In single‑cell data from smoking macrophages, ATF3 also correlated with GEM (r = 0.472, Figure S8C), suggesting transcriptional activation. QPCR analysis revealed that CSE dose‑dependently induced ATF3 in both macrophage models (Figure 7B,C). At single‑cell resolution, GEM expression was higher in macrophages from current smokers than in those from never smokers and former smokers (Figure S8D). Dual‑luciferase assays confirmed that reporter constructs containing the GEM TSS responded to ATF3 overexpression with increased activity in both THP‐1‐M and MH‐S cells (Figure 7D,E), establishing direct transcriptional regulation. In addition, overexpression of ATF3 in both types of macrophages led to a significant increase in GEM mRNA and protein levels (Figure 7F–H).

At the post‑transcriptional level, we examined the m^6^A‑mediated control of GEM. Single‑cell data revealed that GEM expression correlated with the expression of multiple m^6^A regulators (writers/readers/erasers), notably IGF2BP1/2 and FTO, in smoking macrophages (Figure S9A). In GSE130928, GEM was also correlated with several m^6^A regulators, with the strongest and most consistent association observed for the m^6^A reader IGF2BP2 (r = 0.534, Figure 7I; Figure S9B). IGF2BP2 expression was greater in macrophages from current smokers than in those from never smokers or former smokers (Figure S9C). qPCR revealed dose‑dependent induction of IGF2BP2 by CSE in both macrophage models (Figure 7J,K). SRAMP predicted several high‑confidence m^6^A sites on mature GEM RNA (Figure S10). MeRIP confirmed m^6^A enrichment at two predicted sites on GEM transcripts in THP1‑M and MH‑S cells (Figure 7L,M), indicating m^6^A‑dependent post‑transcriptional regulation. Functionally, IGF2BP2 overexpression increased GEM mRNA and protein expression in both macrophage models (Figure 7N–P) and significantly prolonged the GEM mRNA half‑life under transcriptional blockade with actinomycin D (Figure 7Q,R), which is consistent with m^6^A‑guided stabilization by IGF2BP2.

Together, these findings delineate the dual control of GEM upregulation in CSE‑induced macrophage senescence: CSE elevates ATF3, which directly activates GEM transcription, and IGF2BP2 recognizes m^6^A sites on GEM transcripts to stabilize its mRNA.

## 4 Discussion

Most prior work on smoking‐driven diseases has focused on epithelial or endothelial cells. Here, we extend this framework to alveolar macrophages at single‐cell resolution. As the most abundant frontline innate effector cells in the lung, macrophages are central to pulmonary homeostasis, pathogen clearance, and postinjury repair [25]. Macrophage senescence can impair immune function and ultimately contribute to age‐related diseases and malignancy [26]. Many functions of alveolar macrophages are dysregulated after exposure to cigarette smoke [7]. Using human BALF single‐cell transcriptomics, complemented by an in vitro CSE exposure model and public transcriptomes, we delineated the senescence landscape of smoking‐associated pulmonary macrophages. In vitro, CSE induces a conserved cascade across species—ROS accumulation, DNA damage, and activation of the senescence program—accompanied by increased apoptosis and impaired phagocytosis. Together, these clinical and in vitro data support a causal chain linking smoking to macrophage senescence and dysfunction. These findings provide mechanistic insight into smoking‐driven chronic inflammation and lung remodeling and point to translational biomarkers and therapeutic targets.

Integrative analysis across three datasets revealed that GEM is a key mediator of smoke‐induced macrophage dysfunction. GEM expression was greater in smokers than in nonsmokers and correlated with CDKN1A, and our functional data support a driver role in macrophage senescence. Mechanistically, GEM is an RGK GTPase that binds the CaVβ subunit of voltage‐gated Ca2+ channels, reducing channel trafficking and opening probability and thereby reshaping cellular Ca2+ homeostasis and excitation–secretion coupling [27, 28, 29]. GEM also complexes with and inhibits ROCK, remodeling the actin cytoskeleton (stress fibers, focal adhesions, morphology) [28]. In immune cells, Ca2+ influx and cytoskeletal dynamics regulate phagocytosis, mitochondrial function, inflammasome assembly, and receptor signaling microdomains, positioning GEMs to reprogram macrophages [30]. Perturbations in Ca2+ balance and cytoskeletal tension promote mitochondrial ROS and the DNA damage response, activate the p53/p21 and p16INK4A pathways, and cooperate with NF‐κB/STAT3 networks to sustain the SASP [31, 32, 33]. Thus, GEM‐driven remodeling of Ca2+ signaling and the cytoskeletal/mechanical milieu provides a concise mechanistic basis for initiating and maintaining a macrophage senescence–SASP program.

Mechanistically, our findings support a GEM–SIRT3–mitochondrial ROS axis that drives macrophage senescence under cigarette smoke exposure. GEM is inversely associated with SIRT3 and suppresses SIRT3 expression in macrophages. Loss of SIRT3 weakens mitochondrial antioxidant defense, decreases ATP production, and increases mitochondrial ROS. This redox stress stabilizes p21/p16‑dependent cell‑cycle arrest and augments SASP output, thereby reinforcing the senescent state and impairing effector functions. These observations align with prior work showing that SIRT3 is a key mitochondrial deacetylase that maintains redox homeostasis [34, 35] and protects against chronic and acute lung injury, including age‐associated lung fibrosis and endotoxin‐induced acute lung injury [36, 37]. Moreover, mitochondrial dysfunction is a proximal trigger for senescence and SASP activation [38]. Causality is supported by rescue experiments. Pharmacologic activation of SIRT3 suppresses GEM‑induced mitoROS, restores SOD2, reduces SA‑β‑gal positivity, normalizes the cell‑cycle profile, and decreases MMP1 and IL‑1β. We further delineated the mechanisms underlying GEM upregulation in smoke‐exposed macrophages. CSE induces the transcription factor ATF3, which directly enhances GEM promoter‐driven transcription. In parallel, the m^6^A reader IGF2BP2 recognizes m^6^A sites on GEM transcripts and stabilizes their mRNAs, amplifying and sustaining GEM expression. Recent work has shown that RNA modifications regulate the DNA damage response (DDR) and genome stability; these mechanisms shape cellular senescence and disease progression and offer therapeutic entry points [39]. This dual regulation at the transcriptional and posttranscriptional levels provides a unified explanation for sustained GEM upregulation, downstream SIRT3 downregulation, and progression of the senescence program.

This study identifies a GEM–SIRT3–mitochondrial ROS axis as a core mechanism of smoke‐driven macrophage senescence and dysfunction. Smoking upregulates GEM, likely via ATF3‐ and IGF2BP2‑mediated m^6^A regulation, which suppresses SIRT3, elevates mitochondrial ROS, and stabilizes the p21/p16 program and SASP. These findings suggest that GEM, SIRT3, and mitochondrial redox metrics are actionable biomarkers and targets for precision therapy in smoking‑related lung disease.

## Author Contributions

**Jin Wang**: Conceptualization, Methodology, Supervision, Project Administration, Funding Acquisition, Writing – Review & Editing; **Meidan Wei**: Conceptualization, Methodology, Investigation, Data curation, Formal Analysis, Visualization, Writing – Original draft; **Xiangrong Song**: Conceptualization, Methodology, Investigation, Validation, Visualization, Writing – Original draft; **Jiaxin Zhang**: Investigation, Validation, Data curation, Formal Analysis; **Lisha Zhang**: Investigation, Resources, Validation; Sixian Chen: Investigation, Visualization, Data curation; **Yaoyu Hu**: Software, Formal Analysis, Visualization; **Hui Gao**: Resources, Validation; **Jianxiang Li**: Conceptualization, Supervision, Resources, Funding acquisition, Writing – Review & Editing. Jin Wang, Meidan Wei, and Xiangrong Song contributed equally to this work. Corresponding authors: Jin Wang and Jianxiang Li.

## Funding

This study was funded by the China Postdoctoral Science Foundation (Project No. 2023M732527) and the National Natural Science Foundation of China (Project Nos. 82373613 and 82304184). The study was also supported by the Jiangsu Key Laboratory of Preventive and Translational Medicine for Geriatric Diseases, the MOE Key Laboratory of Geriatric Diseases and Immunology and the Priority Academic Program Development of Jiangsu Higher Education Institutions (PAPD).

## Ethics Statement

The study was reviewed and approved by the Institutional Review Board of the Second Affiliated Hospital of Soochow University (Approval No. EC2024625). All procedures involving human participants complied with the Declaration of Helsinki and relevant institutional and national guidelines. The animal study was approved by the Laboratory Animal Ethics Committee of the Experimental Animal Center of Soochow University (Approval No. 202501A014). The study was conducted in accordance with local legislation and institutional requirements.

## Consent to Participate

Written informed consent was obtained from all adult participants prior to enrollment, bronchoalveolar lavage, and data collection. Consent covered access to medical records for verification of smoking history and the use of deidentified clinical and research data.

## Conflicts of Interest

The authors declare that the research was conducted in the absence of any commercial or financial relationships that could be construed as potential conflicts of interest.

## Supporting information




**Supporting File 1**: advs76079‐sup‐0001‐SuppMat.docx.

**Supporting File 2**: advs76079‐sup‐0002‐SuppMat.docx.

## Data Availability

Raw FASTQ files from the human BAL single‐cell RNA‐seq and the THP‐1‐M ± CSE bulk RNA‐seq experiments have been deposited in the NCBI Sequence Read Archive (SRA) under BioProject accession PRJNA1313307.
