# Supplementary material for: Cigarette Smoke‐Induced Alveolar Macrophage Senescence via GEM/SIRT3‐Mediated Mitochondrial Dysfunction
Source: Adv Sci (Weinh). 2026 Jun 22;13(50):e22788. doi: 10.1002/advs.202522788 (PMC13336439; doi:10.1002/advs.202522788)
Supplement: Supplementary file 1 [file ADVS-13-e22788-s001.docx]

1.1 **Cell apoptosis assay**

Cell apoptosis was detected using the Annexin V-FITC/PI Apoptosis Detection Kit (Beyotime, C1062) according to the manufacturer's instructions. Briefly, cells were collected by centrifugation at 1000 rpm for 5 minutes and washed twice with cold PBS. Cell pellets were resuspended in 195 μl Annexin V-FITC binding buffer, followed by the addition of 5 μl Annexin V-FITC and 10 μl PI staining solution. Cells were gently mixed and incubated at room temperature in the dark for 15 minutes. Apoptosis was immediately analyzed by flow cytometry. A minimum of 10,000 events were recorded per sample. Early apoptotic cells were defined as Annexin V-FITC⁺/PI⁻, while late apoptotic and necrotic cells were defined as Annexin V-FITC⁺/PI⁺.

1.2 **Participants and grouping**

We consecutively enrolled adult volunteers aged 40–60 years who underwent diagnostic bronchoscopy with bronchoalveolar lavage (BAL) for evaluation of indeterminate pulmonary nodules at the Second Affiliated Hospital of Soochow University (Suzhou, Jiangsu Province, China). All patients were permanent residents of the Suzhou metropolitan area (predominantly urban); no subjects reported long‑term residence in heavily polluted rural industrial areas. Recruitment and sample collection were performed between July and August 2024, a period with relatively lower coal‑related urban air pollution compared with winter in this region.

Participants were initially classified into two groups (smokers vs. non‑smokers) for the main analyses, with 10 evaluable BAL samples per group (n = 20 total). For more detailed description of smoking exposure, all subjects were further stratified into three categories based on self‑report corroborated by chart review:
(a) current smokers, defined as having smoked within the past 30 days with a cumulative exposure ≥1 pack‑year;
(b) former smokers, defined as having a cumulative exposure ≥1 pack‑year and abstinent for ≥6 months;
(c) never smokers, defined as a lifetime consumption <100 cigarettes and not currently smoking.

Exclusion criteria were: (1) active respiratory or systemic infection (including fever, purulent sputum, or radiologic evidence of pneumonia within the preceding 4 weeks); (2) known systemic autoimmune, autoinflammatory or other chronic immune‑mediated diseases; (3) history of organ transplantation; (4) known HIV infection or other primary immunodeficiency; (5) active malignancy under systemic therapy; and (6) inability or unwillingness to provide written informed consent.

BAL was performed in a radiologically non‑involved segment, using three 50‑mL aliquots of sterile saline with gentle suction. The recovered fluid was filtered through sterile gauze, and cells were pelleted by centrifugation. Total cell counts were determined with a hemocytometer, and cell viability was assessed by trypan blue exclusion. The cell viability across all subjects was range 85.3–94.7%.

Basic demographic and clinical characteristics of the cohort are summarized in Table S1.

1.3 **Single-cell RNA-seq data analysis**

Single-cell RNA-seq data processing and quality control

Fresh bronchoalveolar lavage fluid (BALF) cells from never (n = 3), former (n = 2), and current (n = 3) smokers were processed using the Chromium Single Cell 3′ platform (10x Genomics) according to the manufacturer’s protocol and sequenced on an Illumina NovaSeq 6000 system.

Raw base call files were converted to FASTQ using Illumina bcl2fastq (v2.20). FASTQ files were processed with Cell Ranger (v8.0.1, 10x Genomics) using the “cellranger count” pipeline and aligned to the human GRCh38 reference genome. For each sample, the filtered gene–barcode matrix generated by Cell Ranger was imported into R (v4.5.1) and analyzed using Seurat.

Quality control was performed using standard Seurat metrics. We calculated the number of detected genes per cell (nFeature_RNA) and the percentage of mitochondrial transcripts (percent.mt). Cells were retained if nFeature_RNA < 6,000 and percent.mt < 25. Cells failing these criteria and putative doublets identified by a doublet-detection algorithm were removed. Genes detected in fewer than 3 cells in the entire dataset were excluded from downstream analyses.

Normalization, integration, and dimensionality reduction

Gene expression counts were normalized with Seurat’s “LogNormalize” method (scale factor = 10,000) followed by log-transformation. Highly variable genes were identified using “FindVariableFeatures” with default settings.

To correct for inter‑sample variability, datasets from all donors were integrated using Seurat’s anchor-based integration workflow. Integration anchors were identified with “FindIntegrationAnchors,” and the integrated expression matrix was obtained with “IntegrateData.” The integrated data were scaled and centered, and principal component analysis (PCA) was performed on highly variable genes. The leading principal components were used for downstream clustering and visualization.

Low-dimensional embeddings were generated using t-distributed stochastic neighbor embedding (t‑SNE) and/or uniform manifold approximation and projection (UMAP) as implemented in Seurat.

Clustering and cell-type annotation

Unsupervised clustering was performed using the shared nearest neighbor (SNN) modularity optimization algorithm in Seurat. “FindNeighbors” was run on the selected principal components, followed by “FindClusters” with a resolution parameter chosen to yield a biologically interpretable number of clusters.

Cell types were annotated based on transcriptional profiles, using the expression patterns of established lineage- and cell-type–associated genes together with information from published single-cell lung and immune cell atlases. Expression of representative marker genes was examined by dot plots and heatmaps to confirm the robustness of annotation.

Analysis of senescence-related and SASP-related genes

We focused on the expression of senescence-related genes, including CDKN1A, CDKN2A, and TP53, across major cell types and smoking groups. Average expression levels per cell type and condition were computed and compared, and expression in alveolar macrophages was further assessed at the single-cell level.

To characterize the senescence-associated secretory phenotype (SASP), we curated a panel of SASP-related cytokines and inflammatory mediators based on the literature. Their expression was quantified across major cell populations and smoking groups to evaluate SASP-like changes associated with smoking.

Differential gene expression and enrichment analysis

For macrophage-focused analyses, cells annotated as macrophages were subsetted according to smoking status (current vs. never smokers). Differentially expressed genes (DEGs) were identified using Seurat’s “FindMarkers” function (Wilcoxon rank-sum test) with the following thresholds: absolute log₂ fold change ≥ 0.25, expressed in ≥ 10% of cells in at least one group, and false discovery rate (FDR)–adjusted P < 0.05 (Benjamini–Hochberg correction). The numbers of upregulated and downregulated genes were summarized for each comparison.

Functional enrichment analyses of DEGs were performed using Gene Ontology (GO) biological process and Kyoto Encyclopedia of Genes and Genomes (KEGG) pathway annotations, implemented in clusterProfiler or an equivalent R package. All detected genes were used as the background gene set, and FDR‑adjusted P < 0.05 was considered significant. Enriched terms included immune- and inflammation-related processes, cell adhesion, cytokine responses, and signaling pathways such as TNF, NF‑κB, FoxO, and cellular senescence–associated pathways.

Reclustering and analysis of macrophage subpopulations

Cells annotated as macrophages in the primary analysis were extracted and reanalyzed separately. Following re‑normalization, scaling, and PCA, reclustering was performed using the SNN-based approach with an adjusted resolution to resolve macrophage subpopulations. The resulting subclusters were visualized in low-dimensional space.

Macrophage subclusters were annotated based on their distinct transcriptional signatures and correspondence to previously described macrophage activation states, including inflammation-associated subsets (e.g., IL‑1 receptor–positive and M1-like populations) and SPP1⁺ macrophages. For each sample, the proportions of cells in each macrophage subcluster were calculated and summarized per smoking group to compare macrophage composition among never, former, and current smokers.

1.4 **Mouse cigarette smoking model construction**

Male C57BL/6J mice (8 weeks old, 20–25 g) were purchased from Beijing Vital River Laboratory Animal Technology Co., Ltd. (Beijing, China). The C57BL/6J substrain was selected to avoid mutations present in some other C57BL/6 substrains that may affect inflammatory responses. Mice were housed under specific pathogen free (SPF) conditions in the Laboratory Animal Center of Soochow University (22 ± 2°C, 50–60% humidity, 12 h light/dark cycle) with free access to food and water. All procedures complied with the NIH Guide for the Care and Use of Laboratory Animals and were approved by the Ethics Committee of Soochow University’s Laboratory Animal Center (approval no. 202501A014). Mice were randomly assigned to a cigarette smoke (CS) group and a sham exposed control group (n = 6 per group). Only male mice were used to avoid potential sex related variability in inflammatory and injury responses.

Smoke exposure system and protocol

Cigarette smoke exposure was performed using a custom built whole body active smoke exposure system. Mice were placed in a transparent acrylic exposure chamber (internal dimensions 20 × 30 × 15 cm; volume ~9 L). Mainstream smoke from commercially available Hong Shuangxi (Red Double Happiness) cigarettes, the most widely sold brand in China (11 mg tar, 1.1 mg nicotine, and 13 mg carbon monoxide per cigarette), was drawn continuously into the chamber using a peristaltic pump at a constant flow rate of approximately 0.8–1.0 L/min (continuous draw, no puffing regimen). No dilution air was added. After each cigarette, the chamber was briefly ventilated with room air to prevent excessive accumulation of smoke before the next cigarette.

Each cigarette was completely consumed within 5 min. Mice in the CS group were exposed to six cigarettes per day (three cigarettes in the morning and three in the afternoon), 5 min per cigarette. Exposures were conducted once daily, 7 days per week, for 28 consecutive days. Sham exposed mice were placed in an identical chamber for the same duration and handled in parallel, but exposed to room air only.

Total particulate matter (TPM) concentration in the exposure chamber was monitored using a real time aerosol monitor (TSI-8530), and carbon monoxide (CO) levels were measured using a portable CO analyzer (Testo-315 3). Under the described conditions, TPM in the chamber was approximately 250–350 mg/m³, and CO levels were approximately 250–350 ppm during active smoking periods. Body weight was recorded weekly. Throughout the exposure period, mice were monitored for general health status, including fur condition, activity level, posture, and respiratory rate, and any abnormal findings were documented.

Euthanasia and tissue collection

Twenty four hours after the final smoke exposure, mice were deeply anesthetized with an intraperitoneal injection of sodium pentobarbital (50–80 mg/kg body weight). Once a surgical depth of anesthesia was confirmed (loss of pedal withdrawal and corneal reflexes, absence of response to noxious stimuli), mice were euthanized by cervical dislocation in accordance with approved institutional guidelines. The thoracic cavity was then opened, and the lungs were carefully excised for subsequent bronchoalveolar lavage, fixation, and tissue preservation as described below.
