# Supplementary material for: Cigarette Smoke‐Induced Alveolar Macrophage Senescence via GEM/SIRT3‐Mediated Mitochondrial Dysfunction
Source: Adv Sci (Weinh). 2026 Jun 22;13(50):e22788. doi: 10.1002/advs.202522788 (PMC13336439; doi:10.1002/advs.202522788)
Supplement: Supplementary file 2 [file ADVS-13-e22788-s002.docx]

**Table S1**. Primer sequences used in this study

| Primer | Sequence (5'-->3') |
| --- | --- |
| CDKN1A_F | TGTCCGTCAGAACCCATGC |
| CDKN1A_R | AAAGTCGAAGTTCCATCGCTC |
| CDKN2A_F | GGGTTTTCGTGGTTCACATCC |
| CDKN2A_R | CTAGACGCTGGCTCCTCAGTA |
| GEM_F | GCAACCGCCATTCTGCTAC |
| GEM_R | CTCCCCTATGAGCACCACTC |
| TP53_F | CAGCACATGACGGAGGTTGT |
| TP53_R | TCATCCAAATACTCCACACGC |
| ACTB_F | CAGCACATGACGGAGGTTGT |
| ACTB_R | CTCCTTAATGTCACGCACGAT |
| Cdkn1a_F | CCTGGTGATGTCCGACCTG |
| Cdkn1a_R | CCATGAGCGCATCGCAATC |
| Cdkn2a_F | CGCAGGTTCTTGGTCACTGT |
| Cdkn2a_R | TGTTCACGAAAGCCAGAGCG |
| Gem_F | CCCCTGCAACCTCCGAAAC |
| Gem_R | ACTCCTTGCTCCCCTATAAGC |
| Trp53_F | CCCCTGTCATCTTTTGTCCCT |
| Trp53_R | AGCTGGCAGAATAGCTTATTGAG |
| Actb_F | GGCTGTATTCCCCTCCATCG |
| Actb_R | CCAGTTGGTAACAATGCCATGT |

**Figure S1**. **Bioinformatic analysis of single-cell RNA sequencing (scRNA-seq) data.** (A) Dot plot showing the expression of key marker genes used for cell-type annotation across all clusters identified by unsupervised clustering. (B) Heatmap of fold-change differences in selected senescence-associated secretory phenotype (SASP)–encoding genes across three major cell types in current smokers versus never-smokers. Lollipop plots showing enrichment of differentially expressed genes (DEGs) between macrophages from smokers and never-smokers in the bronchoalveolar lavage (BAL) scRNA-seq dataset for (C) biological processes and (D) signaling pathways. scRNA-seq, single-cell RNA sequencing; SASP, senescence-associated secretory phenotype; DEG(s), differentially expressed gene(s); BAL, bronchoalveolar lavage.

**Figure S2. Subclustering and annotation of macrophage subpopulations in single-cell RNA sequencing (scRNA-seq) data.** (A) t-distributed stochastic neighbor embedding (t-SNE) plot showing the clustering of macrophage subpopulations. Dot plots showing the expression of key marker genes used for cell-type annotation across (B) all macrophage clusters identified by unsupervised clustering and (C) annotated macrophage subtypes. scRNA-seq, single-cell RNA sequencing; t-SNE, t-distributed stochastic neighbor embedding.

**Figure S3. Effects of graded CSE exposure on macrophage apoptosis and phagocytosis.** (A) Representative flow cytometry plots and (B) quantification showing no significant increase in apoptosis rates in THP1-M cells following treatment with increasing CSE concentrations (0%, 2.5%, 5%, 10%) for 24h (n=3 per group), indicating that the observed senescence phenotype is not attributable to cell death. (C) Representative fluorescence images and (D) quantification demonstrating a dose-dependent reduction in phagocytic capacity in THP1-M cells after CSE exposure (n=3 per group), consistent with CSE-induced functional decline. Data shown as mean ± SEM. **, P < 0.01; ***, P < 0.001. CSE, Cigarette Smoke Extract.

**Figure S4. Transcriptomic and functional characterization of CSE-induced senescence in THP1-M cells.** (A) Volcano plot displaying differentially expressed genes (DEGs) in THP1‑M cells following 5% CSE exposure versus control, with significantly upregulated and downregulated genes highlighted. (B) Scatter plot identifying SASP-encoding genes among DEGs, illustrating the transcriptional activation of senescence-associated secretory phenotype components. (C–D) ELISA quantification of MMP1 and IL-1β protein levels in THP1‑M cell supernatants treated with increasing CSE concentrations (0%, 2.5%, 5%, 10%) for 24h (n=3 per group), confirming dose-dependent SASP induction at the protein level; groups not sharing letters differ significantly (P < 0.05). (E–F) Gene Ontology biological process and KEGG pathway enrichment analyses of DEGs, revealing significant enrichment in senescence- and inflammation-related pathways. CSE, Cigarette Smoke Extract; DEGs, Differentially Expressed Genes; SASP, Senescence-Associated Secretory Phenotype.

**Figure S5.** Differential gene expression in macrophages from smokers in GSE130928. (A) Volcano plot showing DEGs between smokers and never-smokers; lollipop plots showing enrichment of DEGs for (B) biological processes and (C) signaling pathways.

**Figure S6.** Scatter plots showing correlations between candidate genes and CDKN1A expression.

**Figure S7.** GEM contributed to the macrophage’s senescence induced by cigarette smoking. qPCR validation of GEM knockdown and overexpression efficiency, (A) THP1-M; (B) MH-S. qPCR of GEM in THP1‑M (C) and MH‑S (D) after treated with different concentrations of CSE. Lollipop plots showing single‑gene enrichment for GEM: biological processes (E) and signaling pathways (F). CCK‑8 analysis of MH‑S proliferation after GEM knockdown (G). qPCR of CDKN1A (H) and CDKN2A (I) after GEM overexpression. Heatmaps showing effects of GEM knockdown on CSE‑induced expression of two senescence‑related genes in THP1‑M (J) and MH‑S (K).

**Figure S8.** Correlation analysis between transcription factors and GEM. (A) Correlations between five predicted transcription factors and GEM expression based on GSE130928. (B) Scatter plots showing the correlation between ATM and ATF3 expression in the GSE130928 dataset. (C) Scatter plots showing the correlation between the expression of ATM and ATF3 in single‑cell macrophage data. (D) ATF3 expression in BALF macrophages from nonsmokers versus smokers, as determined by single-cell RNA sequencing.

**Figure S9.** Correlation analysis between GEM and genes involved in m6A regulation. (A) Correlation heatmap between GEM and m6A regulators in smoking macrophages from single‑cell sequencing data. (B) Scatter plots showing correlations between GEM and genes involved in m6A regulation in GSE130928. (C) IGF2BP2 expression in BALF macrophages from nonsmokers versus smokers from single‑cell sequencing data.

**Figure S10.** Predicted m6A modification sites in mature GEM mRNA using the SRAMP web tool.
